# Genome Structural Variants Shape Adaptive Success of an Invasive Urban Malaria Vector *Anopheles stephensi*

**DOI:** 10.1093/molbev/msaf140

**Published:** 2025-06-06

**Authors:** Alejandra Samano, Naveen Kumar, Yi Liao, Farah Ishtiaq, Mahul Chakraborty

**Affiliations:** Department of Biology, Texas A&M University, College Station, TX, USA; Department of Vector Control, Environmental Surveillance and Disease Ecology, Tata Institute for Genetics and Society, Bengaluru 560065, India; Department of Biology, Texas A&M University, College Station, TX, USA; Department of Vector Control, Environmental Surveillance and Disease Ecology, Tata Institute for Genetics and Society, Bengaluru 560065, India; Department of Biology, Texas A&M University, College Station, TX, USA

**Keywords:** genome structural variation, invasive species, population genetics, insecticide resistance, selective sweep

## Abstract

Global changes are associated with the emergence of several invasive species, although genetic determinants of their adaptive success remain poorly understood. To address this problem, we investigated the role genome structural variants (SVs) play in adaptations of *Anopheles stephensi*, a primary vector of urban malaria in South Asia and an invasive malaria vector in South Asian islands and Africa. Using whole genome sequencing data, we identified 2,988 duplications and 16,038 deletions of SVs in 115 mosquitoes from invasive island populations and four locations from mainland India, the species’ ancestral range. The minor allele frequency of SVs and amino acid polymorphism suggests SVs are more deleterious than the amino acid variants. However, high-frequency SVs are enriched in genomic regions with signatures of selective sweeps, implying a putative adaptive role of some SVs. We revealed three novel candidate duplication mutations for recurrent evolution of resistance to diverse insecticides in *An. stephensi* populations. These mutations exhibit distinct population genetic signatures of recent adaptive evolution, suggesting different mechanisms of rapid adaptations involving hard and soft sweeps helping the species thwart chemical control strategies. We also identify candidate SVs for the larval tolerance to brackish water, which is likely an adaptation in island and coastal populations. Nearly all high-frequency SVs and the candidate adaptive variants in the island populations are derived from the mainland, suggesting a sizable contribution of existing variation to the success of the island populations. Our results highlight the important role of SVs in the evolutionary success of invasive malaria vector *An. stephensi*.

## Introduction

Invasive population expansion of a species often entails rapid adaptation to novel environments (Reznick and Ghalambor 2001). While ecological factors play a role in this process, increasing evidence supports the important contribution of genetic variation to invasive success (Lee 2002). Adaptation over a short evolutionary time relies on mutations conferring large fitness advantages in the new environments (Bomblies and Peichel 2022). Such mutations could arise de novo in the invasive population or through standing genetic variation in the source population (Prentis et al. 2008). Understanding the nature of these mutations and the relative role of novel and preexisting genetic variation in adaptive phenotypes associated with invasive range expansion is critical to predicting the potential for future invasion events. With the recurrent emergence of invasive species across the globe, it has become increasingly important to investigate the genetics of rapid adaptations in such species (Bohlen et al. 2004; Morrison et al. 2004; Hayes et al. 2008).

*Anopheles stephensi* is a primary vector of urban malaria in the Indian subcontinent and Middle East. However, the species is highly invasive and has spread rapidly to new islands, countries, and continents separated by natural barriers. For example, *An. stephensi* has invaded the Indian islands Lakshadweep and the country of Sri Lanka in the last 25 years (Sharma and Hamzakoya 2001; Gayan Dharmasiri et al. 2017; Ishtiaq et al. 2021). Recently, *An. stephensi* was also detected in Djibouti, a country in the Horn of Africa, in 2012 and has since been found in Ethiopia, Somalia, Sudan, and Nigeria (Carter et al. 2018; Kolaczinski et al. 2021; Abubakr et al. 2022; Hemming-Schroeder and Ahmed 2023). Unless controlled urgently, this invasive vector is predicted to spread all over Africa, invading most African countries and putting nearly 126 million people at risk (Takken and Lindsay 2019; Sinka et al. 2020). The threat notice of *An. stephensi* spread issued by the World Health Organization in 2019 further underscores the seriousness of this situation.

*An. stephensi* has adapted to various anthropogenic changes and selective pressures in its native and invasive range in Asia and Africa, making it a formidable obstacle in controlling urban malaria. A primary concern is its resistance to diverse insecticides like DDT, malathion, dieldrin, and deltamethrin in nearly all populations, including South Asia, the Middle East, and Africa (Enayati et al. 2020). Another concern is its adaptation to breed in man-made habitats such as freshwater storage tanks and wells (Sinka et al. 2020). Additionally, *An. stephensi* have been found breeding in brackish water in tsunami-inundated coastal villages on the south coast of India and the recently invaded island country, Sri Lanka (Gunasekaran et al. 2005; Surendran et al. 2019). These adaptations have enabled the rapid range expansion further into urban areas on the Indian subcontinent and the surrounding islands (Gayan Dharmasiri et al. 2017). However, the genomic basis of these adaptations, which play an important role in the invasive spread of this species, remains unknown, impeding effective chemical, ecological, or genetic control strategies.

Genome structural variants (SVs) like duplication, deletion, transposition, and inversion of large (>100 bp) sequences provide a major source of adaptive genetic variation (Daborn et al. 2002; Van’t Hof et al. 2016; Chakraborty et al. 2018; Harringmeyer and Hoekstra 2022). Gene duplications play an important role in the evolution of insecticide resistance in various insect species (Anthony et al. 1998; Newcomb et al. 2005; Zimmer et al. 2018). Metabolic resistance to insecticides can occur through the amplification of detoxification genes such as cytochrome P450s, esterases, or glutathione S-transferases (Liu 2015). For example, duplication and transposable element (TE) insertions in the cytochrome P450 gene *Cyp6g1* are associated with increased resistance to DDT in *Drosophila melanogaster* (Daborn et al. 2002; Schmidt et al. 2010 ). Similarly, a cytochrome P450 gene *Cyp9M10* duplication is linked to metabolic resistance to permethrin in *Culex quinquefasciatus* (Hardstone et al. 2010; Wilding et al. 2012). Resistance can also result from mutations affecting the target sites of insecticides, such as the acetylcholinesterase gene *Ace-1* (Liu 2015). In both *Culex* and *Anopheles* mosquitoes, tandem duplications that pair a wild-type and resistant copy of *Ace-1* lead to resistance to carbamate and organophosphate (OP) insecticides (Labbé et al. 2007; Weetman et al. 2015).

**Fig. 1.**
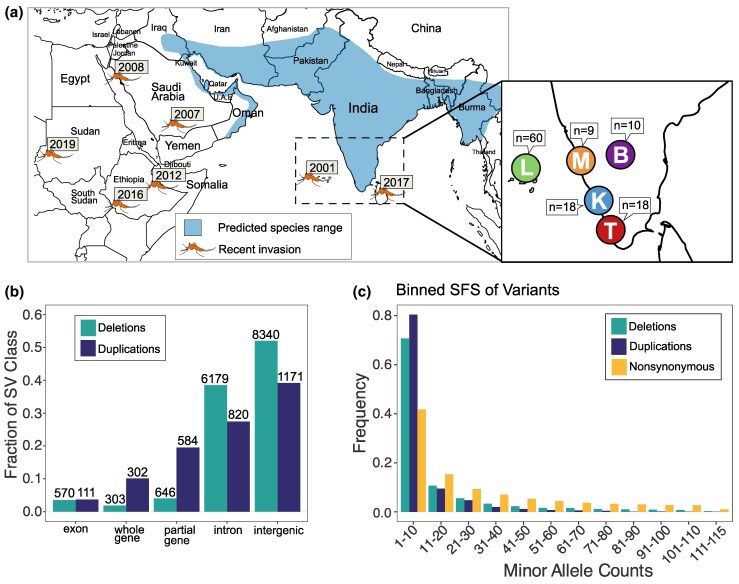
SVs in the Lakshadweep Islands and four mainland populations of *An. stephensi*. a) Predicted distribution and recent invasive spread. Populations examined in this study: Bangalore (B), Mangalore (M), Kochi (K), Trivandrum (T), Lakshadweep (L). The distribution is based on Sinka et al. (2020). b) Distribution of duplication and deletion SVs in five genomic contexts: exonic (fully contained within an exon), whole gene (overlaps at least one complete gene), partial gene (overlaps gene but not completely), intronic (fully contained within an intron), intergenic (fully contained within an intergenic region). c) Binned minor allele counts of duplications, deletions, and nonsynonymous SNPs.

An examination of the population genomics of SVs in *An. stephensi* in native and invasive populations can elucidate the contribution of SVs *to* adaptive evolution and range expansion in this species. However, we only know about a few inversion polymorphisms studied using polytene chromosomes in the South Asian and Middle Eastern populations of *An. stephensi* (Coluzzi 1972; Mahmood and Sakai 1984). Thus, the contribution of SVs in the adaptive evolution of traits relevant to invasion success, such as insecticide resistance or tolerance to brackish water, remains unknown. To investigate the adaptive significance of SVs in *An. stephensi*, we analyzed whole genome sequence data from 115 individual mosquitoes from four mainland locations in India and an archipelago where *An. stephensi* recently invaded, similar to its invasion of Africa (Fig. 1a). Using a population genomic map of duplications, deletions, and TE insertions, we show that SVs provide a significant source of putative adaptive genetic variation in mainland and island populations. We further show that most SVs, including the candidate adaptive SVs, in the island populations were shared with the mainland populations. We further highlight several adaptive copy number variants that are candidates for driving rapid adaptations in the ancestral and the new *An. stephensi* populations in India.

## Results

### Landscape of SVs in *An. stephensi* Populations

To construct the genome-wide map of duplications and deletions, we mapped 150 bp paired-end Illumina reads (average coverage 19.97 assuming a genome size of 235 Mbp, st. dev. 3.31) from 115 individual wild-caught *An. stephensi* mosquitoes from five populations (supplementary fig. S1, Supplementary Material online) to the *An. stephensi* reference genome (Chakraborty et al. 2021) (supplementary table S1, Supplementary Material online). Short reads often miss duplications and report false positives (Chakraborty et al. 2018). Thus, we investigated the genotyping strategy to minimize false negative and false positive rates. We found that the SV detection strategy based on read pair orientation and split-read mapping implemented in the software Delly (Rausch et al. 2012) produced a reasonably reliable map of duplication and deletions for *D. melanogaster* genomes (supplementary fig. S2, Supplementary Material online). We used that approach here for SV detection (see Materials and Methods for details). Additionally, using publicly available long and short reads for an *An. stephensi* strain, we validated 89% (40/45) duplicates and 91% (41/45) deletions from a randomly selected subset of SVs (see Materials and Methods). This indicates a reasonably low false positive rate in our *An. stephensi* dataset. However, it is important to acknowledge that even comprehensive short-read detection methods have limited power in resolving complex SVs. Because short reads miss many SVs, particularly duplications, our SV dataset likely provides a limited representation of such mutations (supplementary fig. S2, Supplementary Material online) (Huddleston and Eichler 2016; Chakraborty et al. 2018). To assess whether the higher false negative rate of SV detection could explain the observed site frequency spectra (SFS) patterns (Fig. 1b), we simulated missing genotypes in the SNP dataset at varying rates. These simulations showed that while genotyping error affects allele frequency estimates, it does not fully account for the skew in the SV SFS toward lower frequencies (supplementary fig. S3, Supplementary Material online), suggesting that the observed differences between SVs and nsSNPs are not solely attributable to detection biases (Materials and Methods).

In the 115 samples, we discovered 2,988 duplications and 16,038 deletions with respect to the reference genome (Chakraborty et al. 2021), with an average of 196 and 2,105 duplications and deletions per individual (supplementary fig. S4, Supplementary Material online). On average, duplications are longer than deletions (median length of duplications is 2,245 and 316 bp for deletions, Wilcoxon rank sum test, *P*-value < 2.2×10^−16^, supplementary fig. S5, Supplementary Material online), similar to their counterparts in *D. melanogaster* (Emerson et al. 2008). Duplication CNVs are more prevalent on the X chromosome than on the autosomes (proportion test, *P*-value < 2.12×10^−4^), which could be because the X chromosome has a lower effective population size, so selection is weaker, leaving more mutations. The distribution of SVs varies between genic and intergenic regions (Fig. 1b). We classified all SVs into five mutually exclusive groups: intronic, exonic, intergenic, whole gene, and partial gene (Fig. 1b, supplementary tables S3 and S4, Supplementary Material online) (see Materials and Methods). Among the SVs, 3.2% overlapped complete genes, 46.8% involved partial genes, and the rest were in intergenic sequences (supplementary table S4, Supplementary Material online). SVs involving partial genes and contained in exons are significantly depleted, which indicates that these are strongly deleterious and are eliminated from these regions by purifying selection (Fisher's exact test, *P*-value < 2.2×10^−16^ − 3.45×10^−8^). We observed a smaller proportion of whole and partial gene deletions than duplications, consistent with the loss of a gene being more deleterious than copying it (proportion test, *P*-value < 2.2×10^−16^).

About 5% (883/16,921) of the deletions completely overlapped annotated TE sequences in the reference genome, suggesting these deletions could represent polymorphic TE insertions in the reference genome (supplementary fig. S6 and S7, Supplementary Material online). The abundance of polymorphic TE insertions in a single genome is similar to that of *D. melanogaster* (Cridland et al. 2013; Chakraborty et al. 2019), implying an extensive structural genetic variation in *An. stephensi* populations due to TE activities. Only 5 TE insertions are in exons, consistent with the harmful effects of TEs disrupting genic sequences. However, 32.16% (284/883) of the TE insertions are in introns, some of which could affect gene expression (Cridland et al. 2015). For example, a 203 bp long terminal repeat (LTR) Gypsy retrotransposon fragment in the first intron of *Ace-2*, a sex-linked paralog of the insecticide resistance gene *Ace-1* (Hemingway and Ranson 2000), is segregating at high frequencies (54% to 89%) in all populations (supplementary fig. S8a and b, Supplementary Material online). A 13 bp sequence (AAACTATAGATCC) present on either side of the TE fragment likely resulted from a target site duplication following the insertion of the fragment (supplementary fig. S8c, Supplementary Material online). The short LTR fragment could be a remnant of a full-length Gypsy element, much of which has been deleted by accumulating indels or recombination between LTRs at both ends of the TE. The first intron of a gene is often enriched with regulatory sequences (Park et al. 2014). LTR TE fragments proximal to a gene can upregulate its expression (Daborn et al. 2002; Chakraborty et al. 2018). Thus, the LTR fragment might alter *Ace-2* gene expression.

### Natural Selection on SVs

To understand the selective forces acting on the SVs, we compared the minor allele frequency spectra of duplications and deletions with that of nonsynonymous single nucleotide polymorphisms (nsSNPs). Nonsynonymous SNPs (nsSNPs) change proteins and are considered deleterious on average (Huber et al. 2017). The allele frequencies of SVs are skewed more toward low frequency than nsSNPs (*P*-value 2.2×10^−16^, χ^2^ test between frequency of SVs and nsSNPs), suggesting stronger purifying selection acting on the SVs on average than the nonsynonymous SNPs (Fig. 1c). However, many SVs (166 duplicates and 2,543 deletions) segregate at a high or intermediate frequency (>0.25) and could include candidates for mutations evolving under positive or balancing selection (supplementary fig. S9, Supplementary Material online). Interestingly, we observed a two-fold enrichment of complete gene duplications in this subset of SVs compared to the genome-wide proportion (19.3% of >25% frequency vs. 10.1% of all duplicates, Fisher's Exact Test, *P*-value 6.2×10^−4^). This enrichment could be due to the higher probability of full gene duplicates having a beneficial function than partial genes or noncoding intergenic sequences. Among the high- and intermediate-frequency full gene duplications, 25 involved protein-coding genes, and 7 encompassed long non-coding RNAs (lncRNA). Whole gene deletions are generally more harmful than complete gene duplicates. Consistent with this, we identified only eight complete gene deletions among SVs segregating at allele frequencies >25%.

Allele frequency of a mutation could rise due to positive selection but also increase due to neutral evolutionary processes. A signature of a selective sweep (Nielsen et al. 2005; Stephan 2019) at or near a high-frequency SV can further support its adaptive significance. To investigate SV mutations that evolved under positive selection, we examined signatures of a selective sweep using composite likelihood ratio (CLR) (Nielsen et al. 2005; DeGiorgio et al. 2016) near SVs in each of the five populations (supplementary fig. 10, Supplementary Material online). Genomic windows with high CLR values are likely to contain adaptive variants. Thus, we examined the abundance of high (>25%) frequency SVs at genomic windows with the top 5% genomewide CLR values. We found SVs segregating at 25% or above allele frequencies are enriched (*P*-values 1×10^−5^ to 0.045) at these 5-kb windows with high CLR values (supplementary fig. 11, Supplementary Material online).

**Fig. 2.**
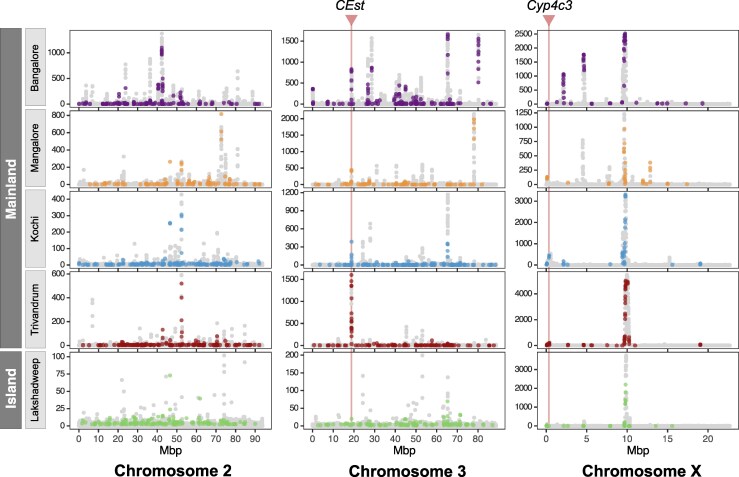
The distribution of CLR statistic from the genome-wide scans for selective sweeps using SweepFinder2. Points are colored if the window CLR is in the 95th percentile and overlaps an SV with an allele frequency greater than 25% in that population. High-frequency duplications of carboxylesterases (CEst) and cytochrome P450 (*Cyp4c3*) genes are associated with CLR peaks in all populations.

Notably, the value of the CLR statistic across the genome is lower for Lakshadweep than the mainland populations, which is likely due to the effects of population bottleneck and growth on CLR (Nielsen et al. 2005; Alachiotis and Pavlidis 2018). However, approximately 43% (877 out of 2050) of the 5 kb windows in the top 5% on the island also show an elevated likelihood of a sweep in at least one mainland population. Gene ontology (GO) enrichment analysis of genes affected by SVs associated with CLR peaks reveals several overrepresented terms associated with insecticide resistance, such as oxidoreductase activity, heme binding, and tetrapyrrole binding (supplementary fig. S12, Supplementary Material online). A prominent peak on the X chromosome is apparent across all populations, which could indicate a strong selective sweep (Fig. 2). Several SVs were detected in this region, most of which are intergenic or affect genes with unknown or unclear function in mosquitos. F or example, high-frequency deletions and polymorphic TE insertions affect a lncRNA and a glutamate receptor-interacting protein (GRIP) within this X chromosome peak (supplementary fig. S13, Supplementary Material online). While the function of the lncRNA remains uncharacterized, a *Drosophila* GRIP homolog organizes muscle guidance and could thus have a role in muscle development (Swan et al. 2004). Notably, an SV near the X chromosome CLR peak involves a partial duplication of the *Cyp9f2*, a gene linked to insecticide resistance (Derilus et al. 2023 ). However, this duplication occurs at intermediate frequencies (0.3 to 0.4) in three of the five populations, suggesting it is unlikely to account for the strong sweep signal observed across all populations. Some SVs exhibit population-specific signals of selection likely indicative of local adaptations (Fig. 2). For example, around the 4 Mbp region on the X chromosome in the Bangalore population (Fig. 2), a high-frequency (0.8) 182 bp deletion is found in *eye-specific diacylglycerol kinase*, a gene essential for photoreception in the *Drosophila* retina. Other prominent, population-specific peaks associated with SVs were examined and found to occur in intergenic regions or uncharacterized genes; thus, we could not determine their adaptive significance. We also identified several high-frequency (>0.8) nsSNPs associated with CLR peaks. These SNPs are within genes implicated in insecticide resistance (*Cyp9f2*), olfaction (*Nrf-6*), and immune response (*CD81*) (Ahmed et al. 2021; Derilus et al. 2023; Heilig et al. 2024) (supplementary table S8, Supplementary Material online).

### Adaptive Evolution at Insecticide Resistance Genes

**Fig. 3.**
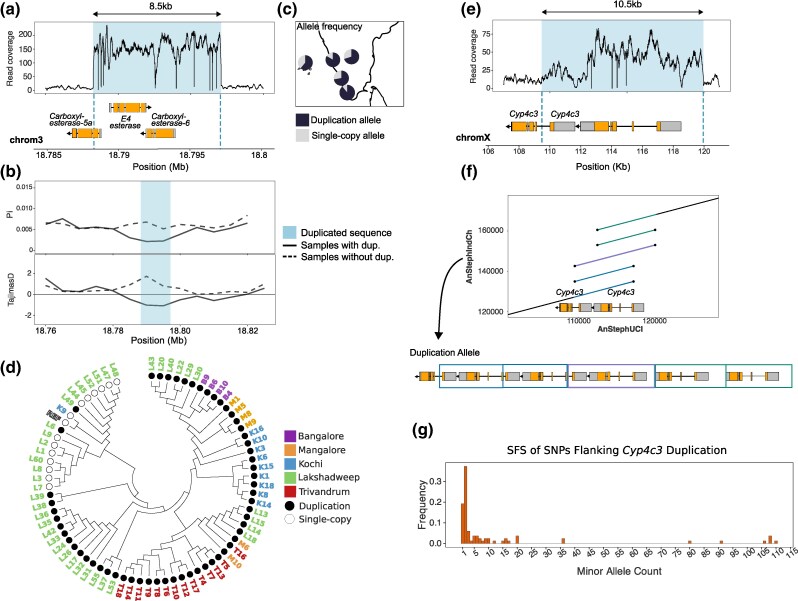
Candidate adaptive duplications for driving widespread insecticide resistance in *An. stephensi.* a) A duplication shown by the coverage of short reads mapped to a cluster of carboxylesterase (*CEst*) genes. b) Reduced nucleotide heterozygosity (π) and Tajima's D flanking the duplication are indicative of recent positive selection. c) Allele frequency of *CEst* duplication allele in five populations. The duplication allele is high frequency in all populations. d) A gene tree constructed from the 20 kb sequences flanking the *CEst* duplication. Only samples homozygous for the single-copy or duplicate allele are shown. All duplicate alleles except two form a single group consistent with a single origin of the duplicate allele. Two duplicate alleles clustering with the non-duplicate alleles likely represent recombination events. e) A duplication of two cytochrome P450 genes is fixed in all populations. f) A dot plot alignment between UCI reference assembly at *Cyp4c3* genes and duplication allele in the IndCh assembly. Lines represent an alignment between the genomes. Colored lines mark the copied sequences which are assembled to show the resulting gene structure of the duplicated region. g) A SFS of minor alleles for SNPs in the 20 kb flanking regions of the *Cyp4c3* duplication.

Indian populations of *An. stephensi*, similar to other Asian and African populations, show widespread resistance to malathion and various pyrethroids (Enayati et al. 2020). Although the increased activity of beta carboxylesterase enzymes is thought to be responsible for this resistance, mutations underlying this enhanced detoxification activity are unknown (Ganesh et al. 2002; Prasad et al. 2017). One duplication SV we identified copies an 8.5 kb sequence on chromosome 3, completely overlapping two beta carboxylesterase genes (Fig. 3a). Based on the read-depth analysis, we estimated the duplicate allele harboring a staggering 15 copies of the genes, suggesting the origin of an insecticide resistance gene array (Chakraborty et al. 2021). This high-frequency duplicate allele (B: 0.7, M: 0.83, K: 0.72, T: 0.89, and L: 0.61) overlaps a high CLR peak on chromosome 3 in all populations, suggesting that the duplicate allele experienced a selective sweep in the recent past (Fig. 2). Reduced levels of nucleotide heterozygosity (π) and Tajima's D around the duplicated region in the haplotype bearing the high-frequency duplicate allele, but not the haplotypes carrying the reference allele, further support the evolution of this duplicate under positive selection (Fig. 3b). The sequences near the duplicate allele are identical across populations, except for some rare SNPs, suggesting a single haplotype with the duplicate allele sweeping through the *An. stephensi* populations in India (Fig. 3d). The inferred selection coefficient (*s*) of the selective sweep based on the intensity of selection (*α*) of the CLR peak is 0.15, suggesting a strong fitness advantage of this duplication. Despite strong selective advantage, the duplication is not fixed in any population, suggesting a recent origin of the mutation. Consistent with this prediction, an estimate of the age of the selective sweep (see Materials and Methods) based on the SNPs flanking the duplicate in the Trivandrum population suggests that the sweep likely occurred very recently, approximately 226.02 generations ago (188.62 to 722.74 generations, 95% confidence interval) (supplementary fig. 14, Supplementary Material online). Amplification of esterases enhances OP insecticide resistance in *Culex* and *Anopheles* mosquitoes (Hemingway and Karunaratne 1998). Thus, the duplication CNV we identified could explain the adaptive increase in carboxylesterase activity in malathion- and deltamethrin-resistant *An. stephensi* strains.

We also identified a duplication CNV of a 10.5 kb sequence near the tip of the X chromosome, which is fixed in all populations and associated with a small CLR peak on the X chromosome (Fig. 2). The duplication copies two cytochrome P450 genes, both of which are similar to *D. melanogaster Cyp4c3*, expressed in the hindgut of feeding larvae (Chung et al. 2009). The low CLR value could be due to an old sweep, which allowed recombination to erode the effect of the sweep on neutral variation linked to the adaptive variant. Due to the proximity of the duplication to the end of the X chromosome, very few SNPs are on the 5′ end of the duplication. However, an absence of the intermediate frequency SNPs on the 5′ and 3′ ends (Fig. 3g) suggests, most likely, a single high-frequency haplotype carrying the *Cyp4c3* duplication reached fixation in all *An. stephensi* populations. A more than 2-fold read coverage indicates the presence of multiple copies of the genes in the duplicate allele. However, uneven coverage across the duplicate suggests different lengths of the individual copies within the duplicate or a heterozygote of two different duplicate alleles being responsible for this pattern (Fig. 3e). To distinguish between these two possibilities, we examined the structure of the *Cyp4c3* duplicate allele in the published genome assembly of an *An. stephensi* strain (IndCh) collected from the southern Indian city of Chennai in 2016 (Thakare et al. 2022). The short-read coverage pattern and breakpoints of the duplicate in IndCh suggest it carries the fixed duplicate *Cyp4c3* allele (supplementary fig. 15, Supplementary Material online). A comparison of the reference and IndCh assemblies at the *Cyp4c3* gene region using a dot plot shows six copies of the gene in duplicate (Fig. 3f). The *An. gambiae* ortholog of *An. stephensi Cyp4c3*, *Cyp4c26*, is overexpressed in pyrethroid-resistant *An. gambiae* strains in Kenya (Bonizzoni et al. 2015), suggesting a role of the *Cyp4c3* duplicate in the reduced susceptibility to pyrethroids like deltamethrin in *An. stephensi* (Tiwari et al. 2010).

While the above examples suggest the involvement of a single duplication allele in all populations, we identified high-frequency gene duplication CNVs in a cluster of epsilon glutathione-S transferases (GSTe), for which two alleles are present in our samples. One allele shares the same breakpoints as a 3.6 kb tandem duplication reported in a laboratory-selected DDT-resistant strain of *An. stephensi* collected in India (Dykes et al. 2022). The duplicate copies two full-length GSTes (supplementary fig. S16a, Supplementary Material online) and is associated with GST overexpression. We uncovered a second allele comprising a 2.9 kb duplication in the same cluster of genes, which also copies two complete GSTes (supplementary fig. S16a, Supplementary Material online). Interestingly, the duplicate reported by Dykes et al. segregates at high frequencies in Bangalore and is absent in Kochi. In contrast, the duplicate we uncovered segregates at high frequencies in Kochi and is lacking in Bangalore (supplementary fig. S16b and c, Supplementary Material online). In Mangalore, Trivandrum, and Lakshadweep, both duplications segregate at intermediate to high frequencies (3.6 kb duplicate 17% to 22% and 2.9 kb duplicate 22% to 53%). The GSTe region does not show a significant CLR peak in any population, likely due to the limited ability of this test to detect sweeps involving multiple haplotypes. To further investigate, we used an alternative approach based on unphased multilocus genotypes (MLGs), the G12 and G2/G1 statistics. High G12 values indicate signatures of selection, while G2/G1 can distinguish between hard and soft sweeps, though our primary focus was on G12 to identify signatures of a soft sweep. We observe elevated G12 at the GSTe cluster in Trivandrum, Kochi, and Lakshadweep, with more pronounced peaks in Kochi and Lakshadweep indicative of a selective sweep (supplementary fig. S16e, Supplementary Material online). The G2/G1 statistic, which compares the frequencies of the two most common MLGs, shows a notable dip in Kochi and Lakshadweep. This reduction in G2/G1 suggests a hard sweep in Kochi, consistent with the observation of only the 2.9 kb duplication. However, it is unexpected in Lakshadweep, where both alleles segregate. Nonetheless, we interpret these results as evidence that this region may have undergone positive selection. The read coverage pattern for 21 samples suggests they are heterozygotes of the two duplicate alleles or carry a recombinant allele. Thus, both GST duplication alleles could be linked to the DDT resistance observed in Asian and African populations of *An*. *stephensi* (Raghavendra et al. 2017; Yared et al. 2020).

### Shared SV Polymorphism in Island and Mainland Populations

**Fig. 4.**
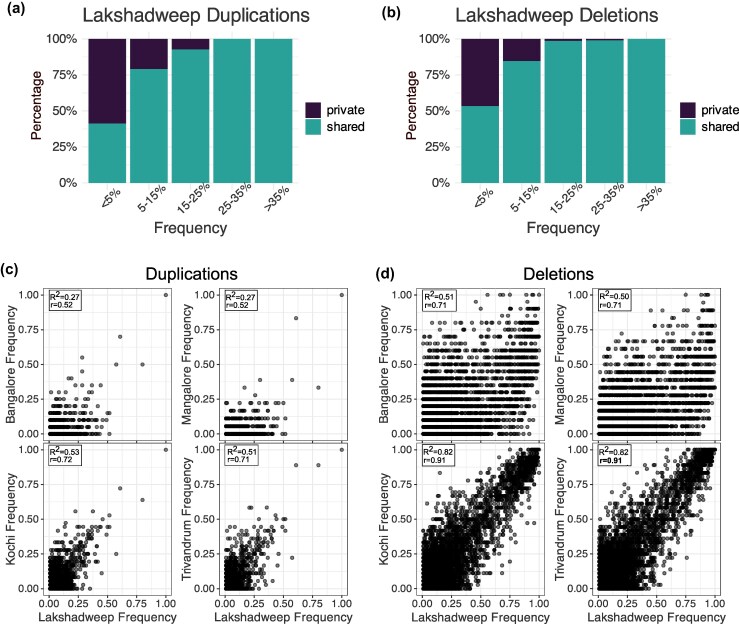
a) Proportion of duplication SVs at different allele frequencies in the invasive island population that are private (found only on the island) or shared with at least one mainland population. Most of the lower frequency duplications are private whereas the higher frequency duplicate SVs are found in both island and mainland populations. b) Proportion of deletion SVs at different allele frequencies that are private to the island or shared with at least one mainland population. As with duplications, deletions that are private to the island segregate at low frequencies. c) Allele frequency of duplications present in the island population is plotted against their frequency in each mainland population. d) Allele frequency of deletions present in the island population is plotted against their frequency in each mainland population. *R*^2^ and *r* represent the coefficient of determination and Pearson correlation coefficient, respectively for the allele frequencies between the island and the mainland populations.

*Anopheles stephensi* invaded Lakshadweep in the last 25 yr, and insecticide resistance and its ability to adapt to a range of larval habitats likely helped the species colonize this island rapidly (Surendran et al. 2019; Ishtiaq et al. 2021). Thus, understanding the source of SVs in the Lakshadweep populations, including the variants contributing to the adaptive traits, can elucidate the relative contribution of segregating and de novo SV mutations in the rapid adaptations of *An. stephensi* in new habitats. In particular, we examined the proportion of SVs in island populations derived from mainland populations. While the specific adaptive mutations in these islands are unknown, such mutations often segregate at intermediate or high (>0.25) frequencies. Nearly all SV mutations in Lakshadweep segregating at frequencies of 0.25 or above are also present in at least one mainland population (Fig. 4a and b). While 0.4% (9,281) of the high-frequency SNPs (>0.25) are unique to the island population (supplementary fig. 17, Supplementary Material online), we found no high-frequency duplications exclusive to the island. Moreover, only 0.2% (4) of high-frequency deletions were private to the island population. Approximately 25% of the SVs segregating at a frequency between 0.05 and 0.25 in the island population are not detected in the mainland populations, suggesting they are private to the island population, or not sampled in the mainland populations. The SV allele frequencies of the island population show a strong correlation with the allele frequencies of SVs from two coastal populations (Kochi & Trivandrum) but not with the SV allele frequencies of the other two mainland (Bangalore and Mangalore) populations (Fig. 4c and d). This pattern could be due to recent divergence between the coastal and island populations, gene flow, similar selective pressures in the two locations, or a combination of these factors. Interestingly, all SVs segregating at high frequency (>0.25) on the island and associated with a sweep window (16 duplications, 239 deletions) are shared with at least one mainland population (supplementary table S9, Supplementary Material online).

Consistent with the mainland being the source of putative adaptive SVs in the island, the esterase, *Cyp4c3, and GSTe* duplicates segregate at high frequencies in Lakshadweep islands (Fig. 3c, supplementary fig. S16b and c, Supplementary Material online). *Anopheles stephensi* populations in coastal regions and recently invaded island populations often show tolerance to brackish water, although mutations responsible for these adaptations remain unknown. We found a 5.5 kb duplication on chromosome 3, which overlaps two larval cuticle protein A2B-like genes and a 1.5 kb intronic deletion in a V-type proton ATPase gene (supplementary fig. S18a and b, Supplementary Material online). Both mutations are present in the island populations as well as coastal populations from Kochi and Trivandrum, where they likely originated. Additionally, the cuticle protein and ATPase genes have been linked to salinity tolerance in *Anopheles* mosquitoes (Smith et al. 2008; Ramasamy et al. 2021; Sivabalakrishnan et al. 2023). Thus, these SVs could alter gene expression of the respective genes and contribute to *An. stephensi* adaptation to brackish water.

## Discussion

Using genome sequencing data from native and invasive populations of the urban malaria vector, *An. stephensi*, we have examined the role of SVs in adaptation to novel environments. We find that SVs present in mainland populations are the predominant source of high- and intermediate-frequency SVs in invasive island populations. Although the SV allele frequency of island populations is driven by a combination of natural selection, ecological connectivity, demography, and gene flow, a high proportion of shared SV polymorphism for the intermediate or high-frequency category suggests that most, if not all, putatively adaptive SVs within this allele frequency category in island populations were derived from the mainland populations. Our finding that 43% of CLR outlier windows on the island are also outliers in at least one mainland population suggests that many candidate loci for selection in the island population were already beneficial on the mainland. While some of these loci may have undergone selection before the population split or share a common selective history, this supports the idea that island adaptation is, at least in part, driven by preexisting beneficial variation. In addition to high-frequency SVs, we observed low-frequency SVs in genes linked to insecticide resistance, host-seeking, mating behavior, and microbial resistance (supplementary tables S5, S6, and S7, Supplementary Material online). These SVs could be a potential source of genetic variation for contemporary or future adaptations.

Our results suggest SVs play an important role in the recurrent and rapid evolution of resistance to various insecticides in *An. stephensi* populations. The candidate duplicates, esterases, *Cyp4c3,* and *GST* likely evolved in the last 70 yr due to the extensive use of insecticides in India after the Second World War. However, whether the timeline of the respective selective sweeps overlapped is unknown. The spread of the esterase duplicate could be driven by malathion, an OP insecticide that was introduced to India in 1969 and continues to be used in indoor residual sprays and outdoor fogging (Batra et al. 2005; Tiwari et al. 2010; Rahi and Sharma 2022). *Anopheles stephensi* has been reported resistant to malathion in six states in India, including Karnataka, where two populations in this study were collected (Raghavendra et al. 2017). However, another possibility is that the spread of this duplication was driven by the more recent introduction of pyrethroid insecticides like deltamethrin. Although pyrethroid insecticide-treated mosquito nets were introduced in India under the National Anti-Malaria Programme in the 1990s (Batra et al. 2005), pyrethroids continue to be used throughout India in ITMNs, indoor residual sprays, and outdoor fogging (Tiwari et al. 2010; Rahi and Sharma 2022). Given our estimation of a very recent selective sweep, it is likely that this duplication spread more recently as an adaptation to the extensive use of pyrethroids and could also explain the cross-resistance to malathion and pyrethroids reported in *An. stephensi*. The duplication overlapping the *Cyp4c3* genes could have been fixed due to the widespread use of synthetic pyrethroid insecticides for malaria vector control. These existing insecticide-resistant alleles from mainland populations would have contributed to the spread of *An. stephensi* in Lakshadweep as insecticides have been used to control malaria in the islands (Sharma and Hamzakoya 2001). Invasive populations of *An. stephensi* in Africa and Sri Lanka have already been found to be resistant to multiple classes of insecticides, suggesting that cross-resistance is a major contributor to the ongoing invasive success of the species. The GSTe duplications, similar to the DDT-resistance *Cyp6g1* alleles in *D. melanogaster*, also show how multiple SV alleles can potentially underlie the evolution of resistance to the same insecticide (Schmidt et al. 2010). In Kochi population, only one duplication allele is high frequency, a hard sweep is suggested by the G2/G1 statistic (supplementary fig. S16e, Supplementary Material online). Surprisingly, this pattern also appears in the populations (Lakshadweep) where both alleles are segregating, and thus, a soft sweep would be expected. Further simulations and a better understanding of *An. stephensi*'s demographic history are needed to elucidate the mechanism of the selective sweep in this population. Notably, the esterase, cytochrome P450, and GSTe gene duplications also show how tandem arrays of functional genes (Shukla et al. 2024) can arise under positive selection.

Our findings are consistent with previous studies in *Anopheles* mosquitoes, including *An. funestus* (Weedall et al. 2020), *An. gambiae*, and other species in the *An. gambiae* complex, including *An. coluzzii* and *An. arabiensis* (Lucas et al. 2019, 2023). These studies show that CNVs affecting metabolic resistance genes can spread through positive selection, driving the widespread insecticide resistance that makes these species significant threats to malaria control. However, unlike earlier findings in *An. gambiae* and *Cx. pipiens* (Labbé et al. 2007; Weetman et al. 2015), we do not observe any full-gene duplications of the acetylcholinesterase gene *Ace-1*. This suggests that gene duplications may play a more significant role in metabolic insecticide resistance than in target-site resistance in *An. stephensi* populations we have investigated.

We also uncovered SVs that may contribute to local adaptation to the environmental conditions in the invasive and coastal populations. A duplication of overlapping cuticle protein genes likely contributes to the larval adaptation to brackish water, which has been observed in *An. stephensi*, as well as other typically freshwater mosquito species. Salinity-tolerant forms of *Ae. aegypti* show increased expression of cuticle proteins, structural changes in larval cuticles, and increased resistance to temephos, an OP larvicide (Ramasamy et al. 2021; Sivabalakrishnan et al. 2023). This duplication may contribute to salinity tolerance in *An. stephensi*, though its role in larvicide resistance is unclear as larval resistance to temephos has not been confirmed in these populations. The deletion in the V-type proton ATPase gene may also contribute to salinity tolerance because changes in the localization of V-type proton ATPase and K+/Na+ ATPase play a role in osmotic regulation in saline-tolerant Anophelines (Smith et al. 2008). The rapid evolution of V-type proton ATPases in the copepod *Eurytema affinis* has been linked to its ability to invade freshwater habitats (Lee et al. 2011), which suggests that changes in this enzyme may contribute to adaptation to different salinities in *An. stephensi*.

Our results show SVs consisting of duplication, deletion, and TE insertions comprise a large proportion of uncharacterized functional genetic variation in *An. stephensi*. Importantly, we show evidence that putative adaptive variants from mainland populations segregate at high frequency in the newly colonized island population. This suggests *An. stephensi* populations invading new geographic locations may benefit from existing adaptive SVs and need not wait for new mutations to adapt to new environmental challenges, which might help *An. stephensi* to spread to new locations rapidly. Resistance to various insecticides and tolerance to diverse osmotic conditions are key adaptations in *An. stephensi* invasive populations in both Africa and Asia. Our results suggest SVs may play an important role in those adaptations. The ability to adapt to new environments is a crucial property of invasive species. Our results from *An. stephensi* suggest SVs can be a potential contributor to that property. However, our SV data based on short reads reveals only a fraction of total SVs in *An. stephensi* (supplementary fig. S2, Supplementary Material online), potentially masking some adaptive variants (Chakraborty et al. 2018). Further research into the functional and fitness role of SVs using long reads in the ancestral and newly colonized *An. stephensi* populations in Asia and Africa, as well as other invasive species, will help us better understand the role of SVs in invasion success.

## Materials and Methods

### Genomic Data Collection

Individual wild-type male and female mosquitoes were collected from four mainland populations and islands from the Lakshadweep archipelago in India (supplementary table S1, Supplementary Material online) (Bangalore *n* = 10, Mangalore *n* = 9, Lakshadweep *n* = 60, Kochi *n* = 18, and Trivandrum *n* = 18). The genomic DNA was isolated from homogenized whole mosquitoes using Qiagen Blood and Tissue kit (Qiagen). Data for the Bangalore and Mangalore strains are from a previously described sample (Thakare et al. 2022). All sequence data was generated using paired-end sequencing performed on an Illumina HiSeq 2500 platform (Illumina, San Diego, CA, United States) at the Tata Institute of Genetics and Society.

### Evaluation of SV Detection Strategies

We performed a benchmark analysis of SV detection methods to evaluate the performance of four short-read SV callers: Delly2 (Rausch et al. 2012), Lumpy (Layer et al. 2014), CNVnator (Abyzov et al. 2011), and Manta (Chen et al. 2016). Paired-end Illumina sequencing data (Shukla et al. 2024) from two inbred *D. melanogaster* lines, A3 and A4, were sampled to 10, 15, and 25 × coverage depth and aligned to the ISO1 reference genome (Hoskins et al. 2015). To simulate a heterozygous individual, we randomly sampled reads from both A3 and A4 to the same three coverage depths. We then applied the four short-read SV callers to identify duplications and deletions. The control dataset was generated using an assembly-based SV caller (https://github.com/yiliao1022/SVGAP) by comparing the highly contiguous genome assemblies of A3 and A4 to the reference ISO1 assembly (Chakraborty et al. 2019). The SVs identified with simulated short-read sequencing data at different depths were then compared with control data to evaluate the true positive rate and positive predictive value of each SV caller.

To evaluate whether genotyping errors in SV calls could account for differences in the SFS between SVs and nsSNPs, we simulated missing genotypes in the SNP dataset at varying rates. Given that SVs typically exhibit a higher false negative rate compared to SNPs, we sought to determine whether this discrepancy could explain the observed skew in the SV SFS. We introduced missing data at different proportions in the SNP dataset, reflecting the expected range of genotyping error rates for SVs. For each level of missingness, we recalculated the SFS for nsSNPs and compared the average SFS from these simulations to the SFS observed for duplications. This approach allowed us to assess whether accounting for genotyping error in SNPs could reproduce the SFS pattern observed for SVs.

### SV Genotyping

We used Trimmomatic v0.36 (Bolger et al. 2014) to remove adapters and trim low-quality regions from reads, followed by FASTQC v0.11.8 to check read quality. Trimmed reads were then mapped to the AnSteph UCI reference genome (Chakraborty et al. 2021) using bwa-mem v0.7.17 with default parameters (Li 2013). Optical duplicates were marked and filtered out using Picard v2.23.9. To identify duplication and deletion SVs segregating in the five populations, we used Delly to implement read-pair orientation analysis (Rausch et al. 2012). We filtered SV calls, keeping only deletion and duplication variants between 100 bp and 100 kb and excluding those with “LowQual” flags using BCFtools v1.14 (Danecek et al. 2021). To merge SV calls across all 115 samples, we used Jasmine v1.1.5, which uses an SV proximity graph to merge variants present in multiple samples (Kirsche et al. 2023). We excluded SV calls with over 90% overlap with reference TEs. Deletion calls with over 90% reciprocal overlap with annotated TEs were considered polymorphic TE insertions and separated from the other deletion calls. The allele frequency of SVs was calculated from the merged call set using VCFtools v0.1.16 (Danecek et al. 2011).

Genotypes for candidate SVs were further inspected to confirm allele frequency and copy number. Copy number for the carboxylesterase duplication was determined from normalized read depth calculated by CNVnator v0.4.1, a coverage-based SV caller (Abyzov et al. 2011). The *Cyp4c3* duplication is reported as two separate, overlapping duplication calls, likely due to the different lengths of duplicated sequences within this mutation. A total of 101/115 individuals are genotyped heterozygous for both duplications. However, visual inspection of this region of the BAM files in IGV (Thorvaldsdóttir et al. 2013) indicated that all samples show the coverage pattern observed in Fig. 3e. Most of the samples incorrectly genotyped by Delly were male and, therefore, have lower X chromosome coverage, which could explain why this duplication was not called for these individuals. We also found several silent mutations within the duplicated sequence and in the flanking regions fixed in all populations, which we used as additional evidence to infer the fixation of this duplication allele in all populations.

### Validation

To evaluate the accuracy of our genotyping methods with *An. stephensi* data, we applied our SV detection pipeline to 20 × Illumina sequencing data from IndCh (Thakare et al. 2022). We randomly selected 45 duplications (15 per chromosome) and 45 deletion SV calls found in both IndCh and our population SV dataset for further inspection. In particular, we checked whether long- and short-read coverage for reads mapped to the UCI reference (Chakraborty et al. 2021) agreed with the filtered Delly calls, which use read-pair orientation and split-read mapping to infer the SVs. We found that 89% of duplications (40/45) and 91% of deletions (41/45) were supported by long and/or short read coverage (supplementary table S2, Supplementary Material online). Some SVs supported by long reads are not supported by short reads and vice versa. This is because the IndCh strain is segregating for multiple haplotypes, and the DNA source for the short and long reads are separate, causing some haplotypes to be represented only in the long or short reads.

### SV Ancestral State Determination

To confirm that a variant corresponds to the derived state, a closely related outgroup species genome is needed to infer the ancestral state. The closest related species to *An. stephensi* with a high-quality genome assembly is *An. gambiae* (Habtewold et al. 2023). We performed pairwise alignment between the *An. stephensi* and *An. gambiae* reference genomes (Chakraborty et al. 2021; Habtewold et al. 2023) using LASTZ v1.04.15 (Schwartz et al. 2003) and identified syntenic blocks using the UCSC Chain/Net pipeline (Kent et al. 2003). 50.44% of the *An. stephensi* genome aligned to *An. gambiae,* but only 5.1% of duplications and 13% of deletions in our callset fall within these syntenic regions. For this reason, we focused on determining the ancestral state of genic SVs, as coding regions are likely to be conserved between the two species, specifically genic SVs segregating at allele frequencies over 25% in a population. We used exonerate v2.4.0 (Slater and Birney 2005) to find ungapped alignments between the protein-coding sequence of completely duplicated or deleted genes and the *An. gambiae* genome. Additionally, we visually inspected syntenic regions using the JBrowse Genome Browser in VectorBase. For gene duplications, if the gene was present in higher copies in *An. gambiae* than the *An. stephensi* reference, the duplication is inferred to be the ancestral state. For gene deletions, if the gene is present in fewer copies in *An. gambiae*, the deletion is inferred to be the ancestral state (a duplication in the *An. stephensi* reference).

### SNP Genotyping

SNPs were identified using FreeBayes v1.3.5 (Garrison and Marth 2012) with the -C flag set to 2, specifying that a minimum of two reads must support the alternate allele to be called and the -0 flag, which excludes partially mapped reads from variant calling. Indels and SNPs that were multiallelic, had QUAL less than 20, or were called in less than 75% of individuals were filtered out, and the remaining 9,223,910 SNPs were annotated to predict their functional effect using SnpEff (Cingolani et al. 2012).

### Population Structure Analysis

To perform principal component analysis for our SNP data, we used PLINK/2.00a3.7 (Purcell et al. 2007). SNPs with minor allele frequency of less than 0.05 were filtered out, and linkage pruning was performed to remove SNPs in linkage disequilibrium. The first and second principal components were plotted in R.

### Detection of Selective Sweeps

To detect genomic regions with signatures of recent positive selection, we used SweepFinder2, which performs genome-wide scans for selective sweeps by comparing the observed patterns of genetic diversity to a null model generated from the genome-wide frequency spectrum (Nielsen et al. 2005; DeGiorgio et al. 2016). The composite likelihood test implemented by SweepFinder2 has been found to have high power in detecting recent selective sweeps while being robust to demographic factors (Nielsen et al. 2005). Using the quality-filtered SNP data, the CLR within each population was calculated in non-overlapping windows of 5 kb. We considered CLR peaks above the 95th percentile as putative selective sweeps. Signatures of selective sweeps can be located within a 10 kb window centered on the true location of the sweep (Nielsen et al. 2005). Therefore, we considered an SV to be associated with a CLR peak if it overlaps or is within 5kb of a putative sweep window (supplementary fig. S9, Supplementary Material online). We calculated Tajima's D and nucleotide heterozygosity (π) for nonoverlapping 5 kb windows using VCFtools v0.1.16 (Danecek et al. 2011). These summary statistics were calculated within each population and between samples with and without candidate SVs.

To further test for genomic signatures of positive selection, we calculated the G12 and G2/G1 statistics, which detect selective sweeps from unphased data by assessing MLGs (Garud et al. 2015; Harris et al. 2018). This method identifies regions dominated by a small number of high-frequency haplotypes. Then, it compares the frequency of the two most frequent MLGs in this region to distinguish between hard and soft selective sweeps. We further examined the region containing the two high-frequency *GSTe* duplicate alleles, as the spread of multiple beneficial alleles could produce a signature of a soft sweep. Because the Bangalore and Mangalore sample size is much lower than recommended for this test, we performed the analysis on the Kochi, Trivandrum, and Lakshadweep populations. G statistics were calculated using analysis windows containing 100 SNPs, with the centers of adjacent windows separated by 50 SNPs. To estimate a significance threshold for G12, we used the highest value obtained from neutral simulations performed with MSMS (Ewing and Hermisson 2010) using demographic parameters inferred by *dadi* (Gutenkunst et al. 2010) for each population (See Inference of Time Since Onset of Selection).

### Enrichment of SVs in Genomic Contexts

All SVs were classified into five mutually exclusive groups: exonic (fully contained within an exon), whole gene (overlaps at least one complete gene), partial gene (overlaps gene but not complete), intronic (fully contained within an intron), intergenic (fully contained within an intergenic region). To determine whether SVs are overrepresented or depleted in these genomic contexts, we compared the observed distribution to a null distribution generated by randomly shuffling the breakpoints of our SV call set 1,000 times using BEDtools v2.30.0 *shuffle* (Quinlan and Hall 2010) following an approach used in previous work (Cardoso-Moreira et al. 2016). We note that this method does not account for variations in SV detection power across the genome, particularly in repetitive regions, though it may provide a reasonable approximation of a null distribution.

### Enrichment of SVs Near CLR Peaks

To determine whether SVs segregating at allele frequencies greater than 25% are enriched near CLR peaks, we randomly shuffled the breakpoints of these SVs using BEDtools v2.30.0 *shuffle* (Quinlan and Hall 2010) and counted the number of SVs associated with a genomic window with CLR values higher than the 95th percentile. We repeated this 100,000 times for each population to generate null distributions. *P*-values were estimated using the following formula *p* = (*r* + 1)/(*n* + 1) (North et al. 2002), where r is the number of replicates in which the number of SVs associated with a CLR peak is greater than the observed number and *n* is the number of replicates.

### GO Term Enrichment Analysis

We used the topGO R package v2.56.0 (Rahnenfuhrer 2024), to perform GO enrichment analysis for genes affected by SVs segregating over 25% in a population and near CLR peaks. We obtained GO annotations for the UCI AnSteph reference genome from VectorBase.

### Inference of Time Since Onset of Selection

We applied an approximate Bayesian computation (ABC)-based approach adapted from Ormond et al. to estimate the time since the onset of selection (Ts) on the high-frequency esterase duplication (Ormond et al. 2016). While the duplication is high frequency in all populations, we used data from one mainland population, Trivandrum, for this calculation. We used one mainland population rather than all populations used in this study to minimize assumptions about the unknown demographic history of *An. stephensi* in India. Because the esterase duplication allele likely originates from a single event, the selective sweep estimate from one population is expected to align closely with estimates from other populations. We note that demographic factors, such as gene flow between populations, could lead to inaccurate estimates of time since the sweep for all populations. However, we chose a simplified framework to minimize the risk of overfitting the analysis due to uncertain demographic parameters. First, we used *dadi* (Gutenkunst et al. 2010) to fit the demography model to the SFS of synonymous SNPs and estimate the model parameters. Based on the likelihood estimates of various demographic models, including two-epoch, three-epoch, and growth models, we found that the best-fitting model was that of a population bottleneck followed by exponential growth. Using the estimated effective population size of 1,757,732 inferred by *dadi*, we obtained an estimate for the strength of selection *s* from the inferred strength of selection by SweepFinder2. In particular, we used the following formula: *s* = *r* × ln(2Ne)/*α* (Durrett and Schweinsberg 2004; Nielsen et al. 2005) where Ne is obtained from *dadi* estimated parameters, the intensity of selection α of the CLR peak overlapping the duplication, and an assumed *r* = 0.01cM/Mbp. Although an inaccurate estimate of the recombination rate could introduce some bias, SweepFinder2 has been shown to be robust to variations in recombination rate assumptions (Nielsen et al. 2005; Alachiotis and Pavlidis 2018). To generate a prior distribution for Ts, 500,000 simulations were run with the coalescent simulation program MSMS (Ewing and Hermisson 2010), drawing Ts from a uniform distribution and incorporating the estimated s and demographic scenario inferred from *dadi*. Polymorphism data for the 20 kb sequences flanking the esterase duplication were used for simulations. Summary statistics for simulations were calculated from MSMS output using the libsequence library (Thornton 2003) msstats function. Using the R package *pls* (Mevik and Wehrens 2007), a partial least squares method was applied to incorporate the most informative summary statistics into the ABC calculation. The posterior distribution for Ts was generated using an ABC rejection algorithm implemented by the R package *abc* (Csilléry et al. 2012). The point estimate for Ts was calculated from the mode of the posterior distribution.

## Supplementary Material

msaf140_Supplementary_Data

## Data Availability

All scripts and intermediate data files, including the merged SV VCF files, necessary to reproduce the work are available at https://github.com/chakrabortymlab/stephensi2024. The annotated SNP VCF is available at https://zenodo.org/records/14542541. The raw reads have been deposited to NCBI under the Bioproject accession PRJNA1242027.
