# Supplementary material for: Genome Structural Variants Shape Adaptive Success of an Invasive Urban Malaria Vector Anopheles stephensi
Source: Mol Biol Evol. 2025 Jun 6;42(6):msaf140. doi: 10.1093/molbev/msaf140 (PMC12198770; doi:10.1093/molbev/msaf140)
Supplement: msaf140_Supplementary_Data [file msaf140_supplementary_data.zip › Supplementary_Material.pdf]

Supplementary information

Genome structural variants shape adaptive success of an invasive urban malaria vector *Anopheles stephensi*

Alejandra Samano[1], Naveen Kumar[2], Yi Liao[1], Farah Ishtiaq[2], Mahul Chakraborty[1]
Affiliations:

[1]Department of Biology, Texas A&M University, USA

[2]Tata Institute for Genetics and Society, inStem building, GKVK Post, Bengaluru 560065, India

Supplementary Figures

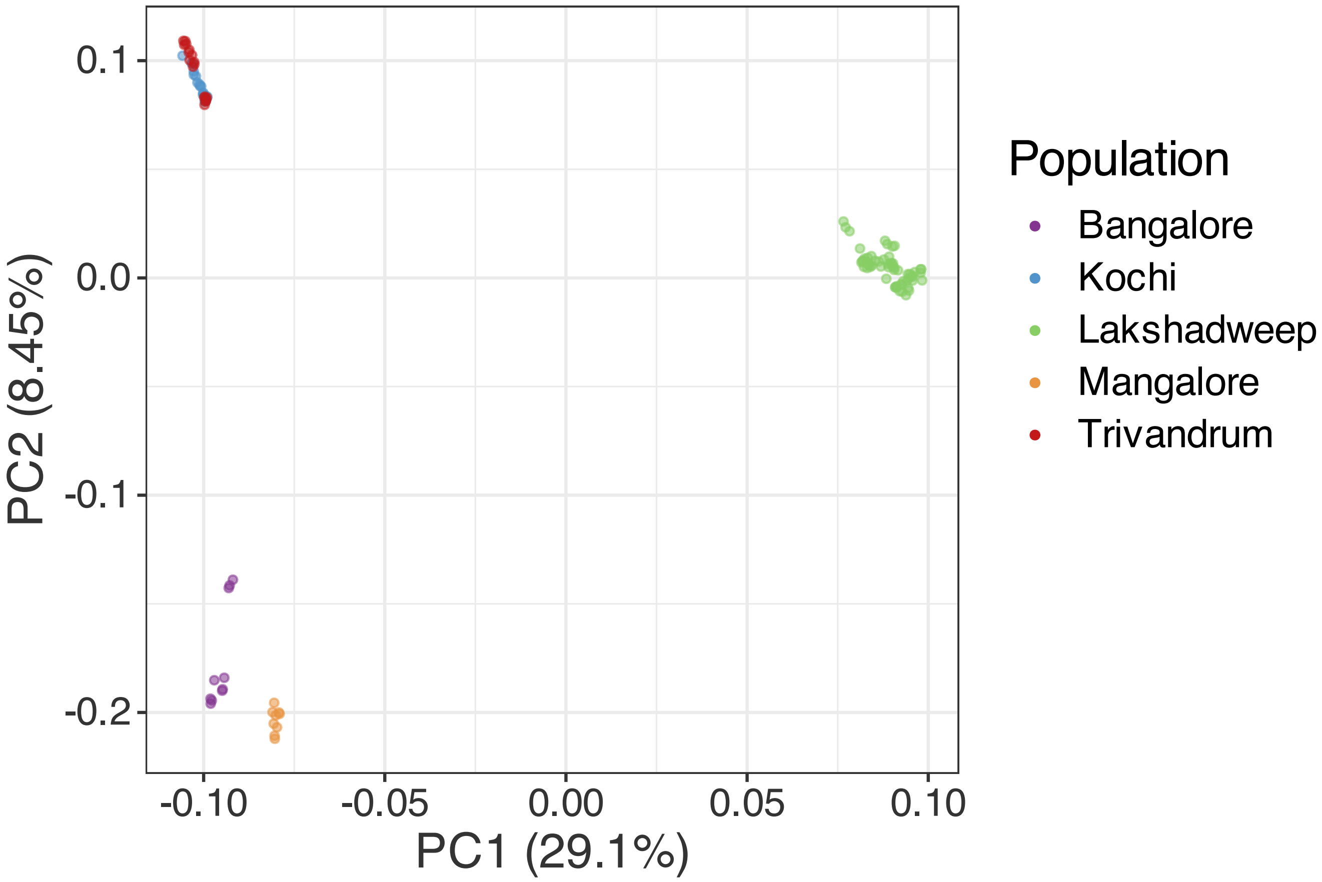

Supplementary Figure 1. Principal component analysis of genome-wide SNPs. The first principal component (PC1), which explains 29.1% of the variance, separates the island population from the mainland populations. The second principal component (PC2), accounting for 8.45% of the variance, distinguishes inland populations (Bangalore and Mangalore) from coastal populations (Trivandrum and Kochi) and the Lakshadweep islands. Notably, Trivandrum and Kochi form a distinct cluster, reflecting a high degree of similarity between these two populations approximately 207 km apart, both located in the state of Kerala. Furthermore, PC2 positions Lakshadweep closer to the coastal populations, suggesting a stronger genetic similarity than the inland populations.

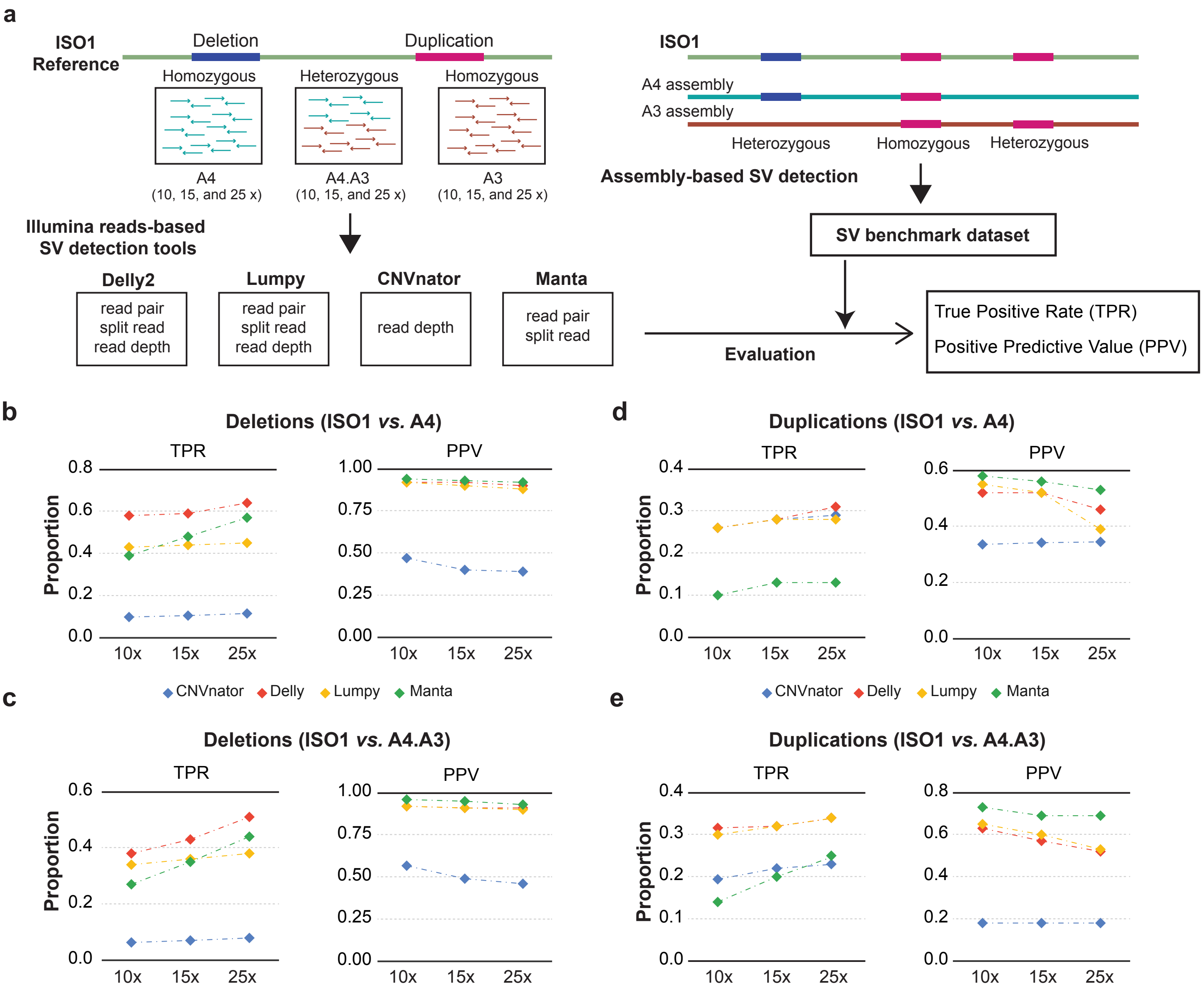

Supplementary Figure 2. Strategy and workflow of the benchmarking analysis. **a.** Paired-end Illumina reads from two inbred *Drosophila melanogaster* lines were sampled at three different coverage depths. Heterozygous data was simulated by randomly sampling reads from two lines. The performance of 4 short-read mapping SV callers was evaluated by comparing them to SV calls from assembly-based SV detection. The true positive rate (TPR) refers to the proportion of total SVs in the assembly discoverable by the short-read caller. The positive predictive value (PPV) refers to the proportion of SVs detected by the short-read caller also present in the assembly. **b.** Evaluation of deletion calls from A4 reads mapped to the ISO1 reference assembly. **c.** Evaluation of deletion calls from simulated A3/A4 heterozygous reads mapped to the ISO1 reference assembly. **d.** Evaluation of duplication calls from A4 reads mapped to the ISO1 reference assembly. **e.** Evaluation of duplication calls from simulated A3/A4 heterozygous reads mapped to the ISO1 reference assembly.

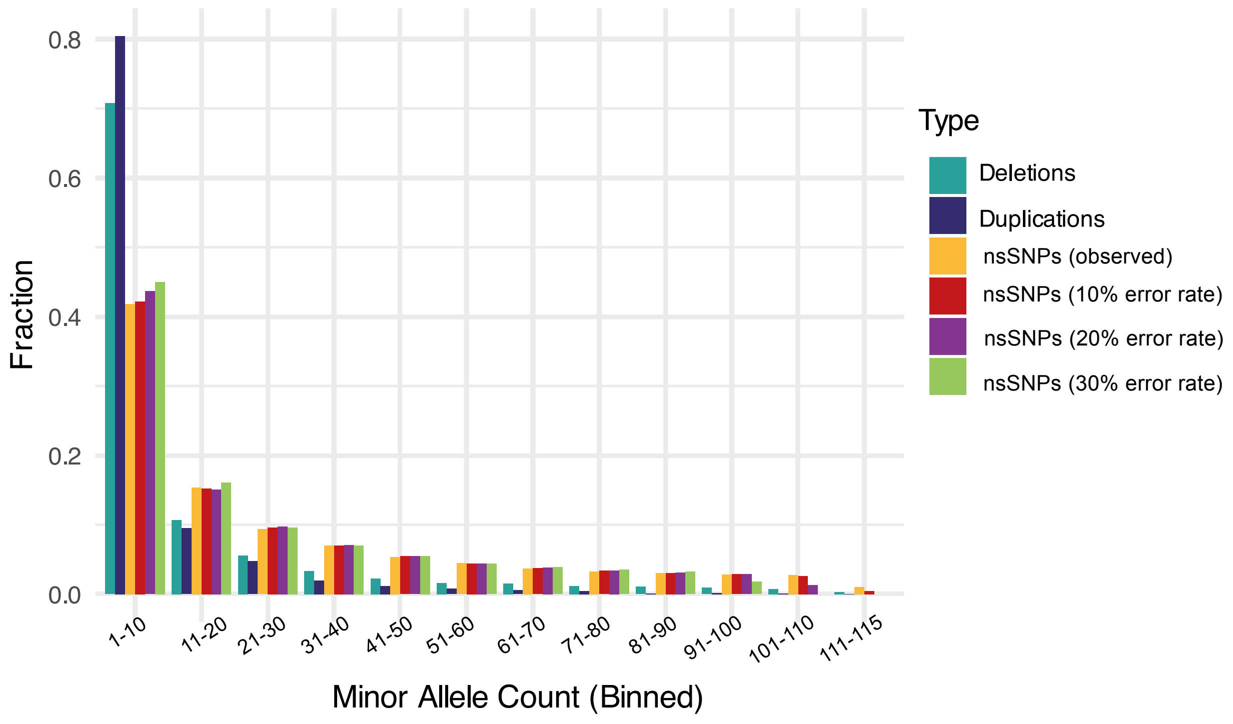

Supplementary Figure 3. Binned site frequency spectrum of SVs, nonsynonymous SNPs, and simulated nonsynonymous SNP datasets with missing data added at different rates.

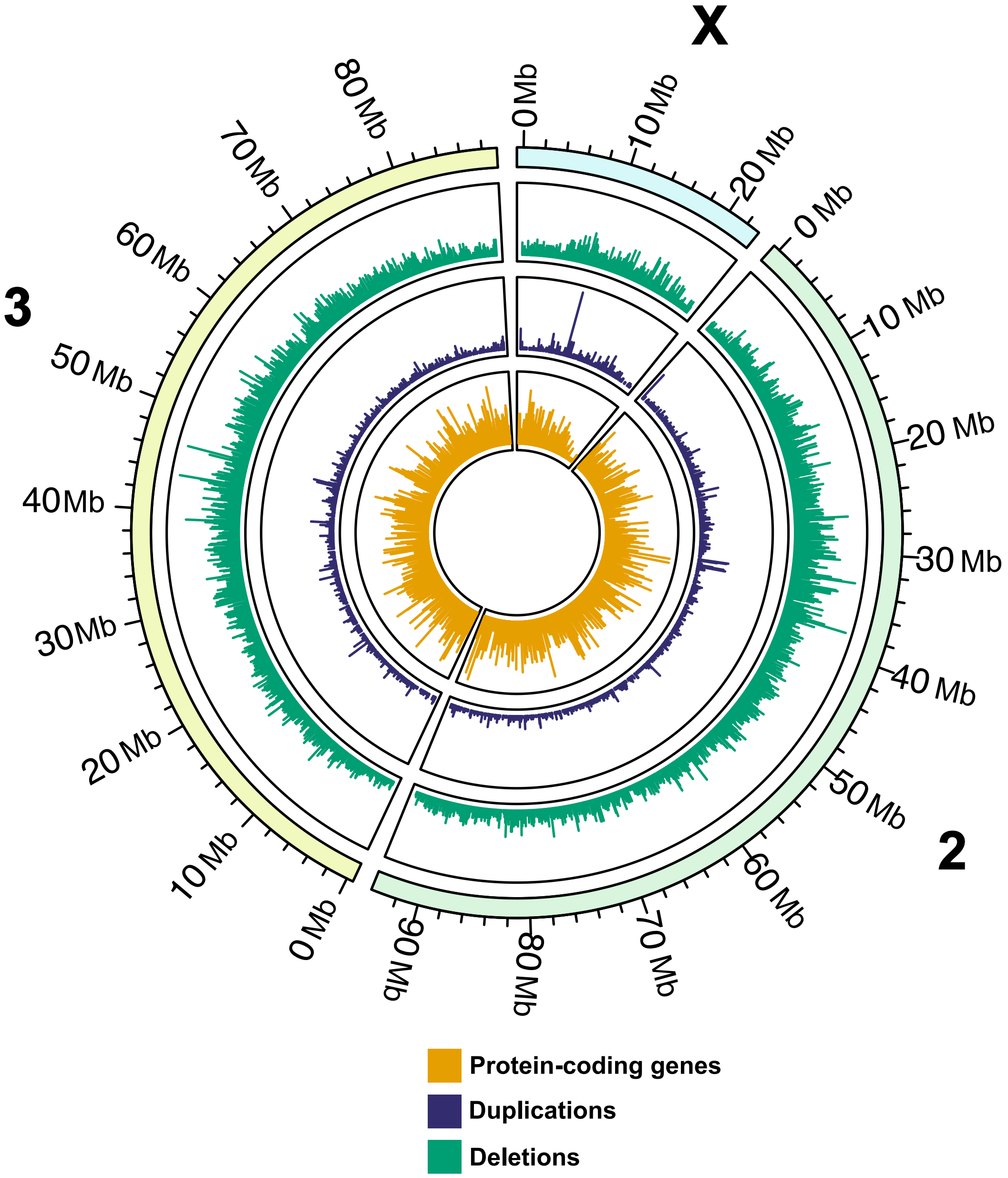

Supplementary Figure 4. Distribution of CNVs (relative to the AnStephUCI reference) of length 100bp-100Kbp and genes across chromosomes 2, 3, and X. Tracks show the number of deletions (green), duplications (purple), and protein-coding genes (orange) per 100 Kbp window in the genome.

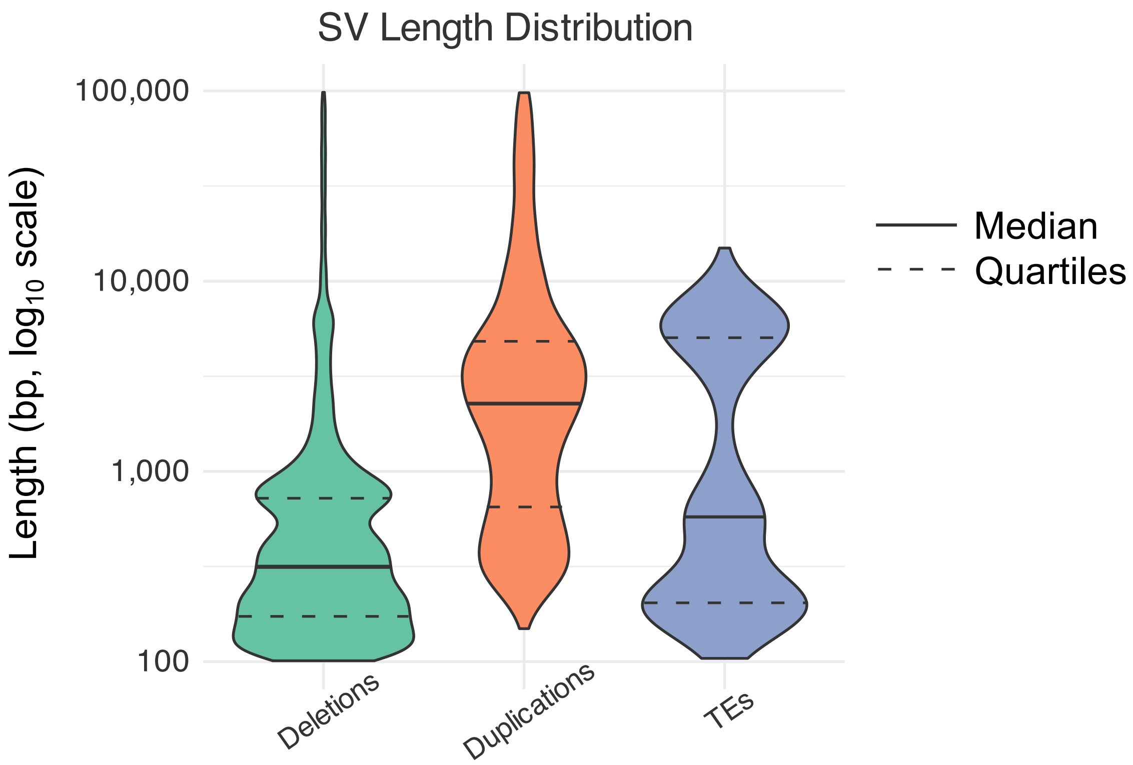

Supplementary Figure 5. Length distribution of deletions, duplications, and polymorphic TE insertions.

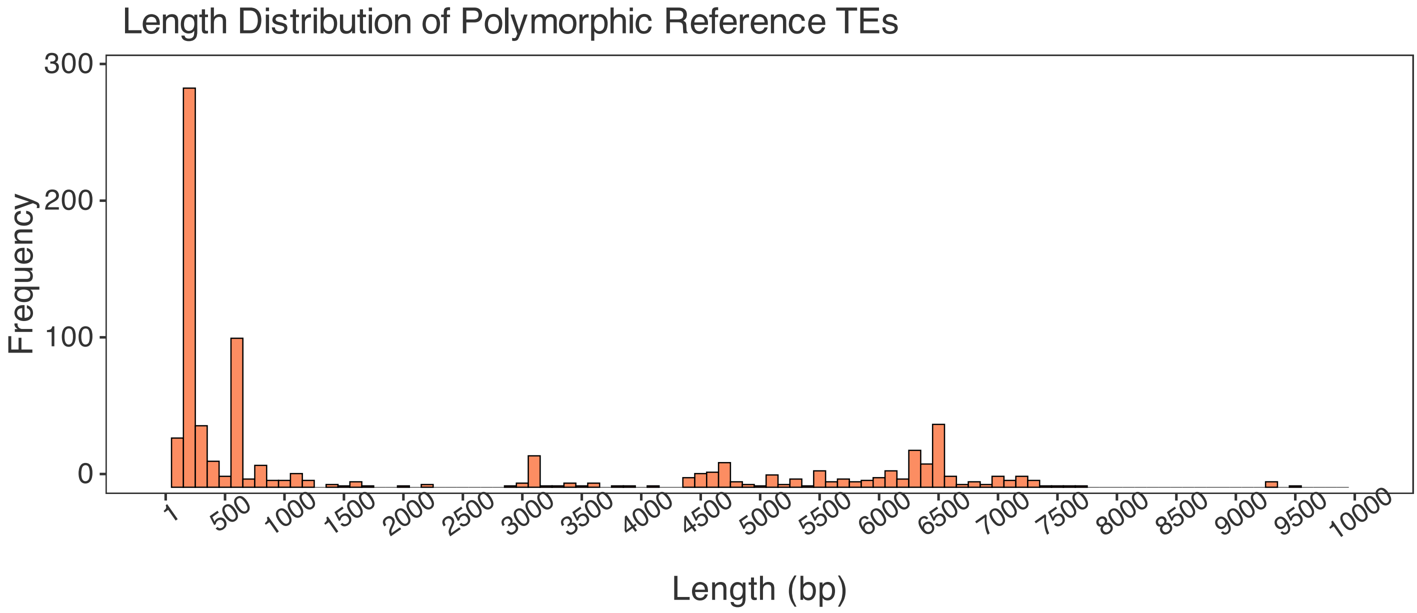

Supplementary Figure 6. Binned histogram of polymorphic reference TE lengths under 10 kb. The peaks near 3 kb, 4.5 kb, and 6.5 kb likely represent lengths of major active full-length TEs in *An.stephensi*.

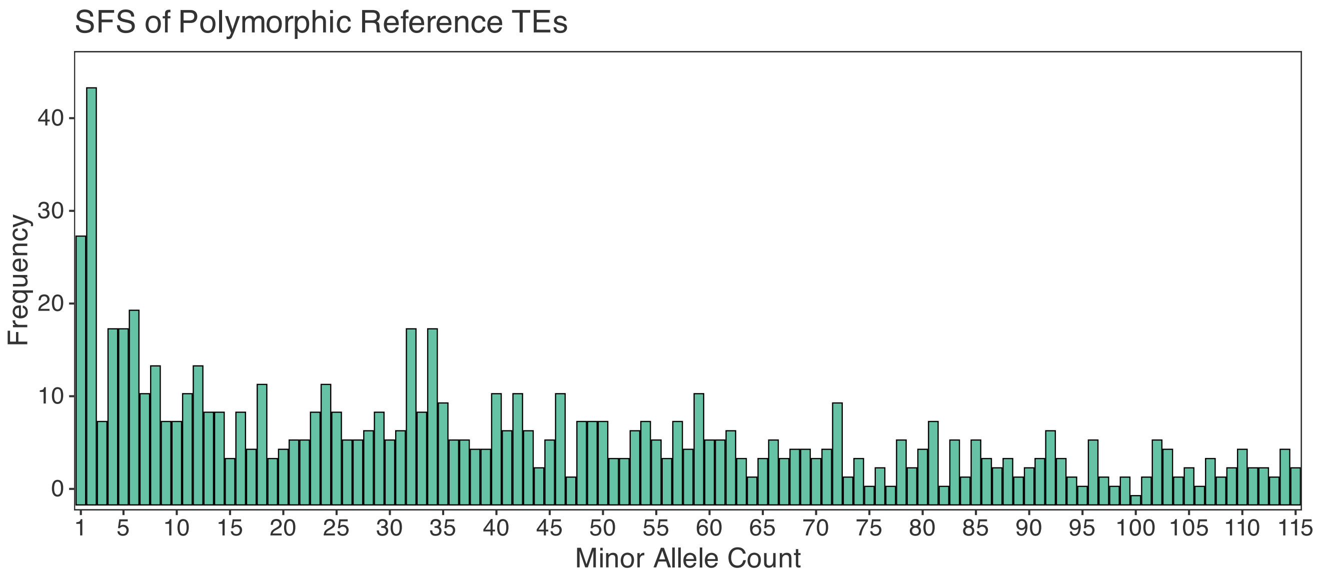

Supplementary Figure 7. Histogram of minor allele counts for polymorphic TE insertions in the AnStephUCI reference genome.

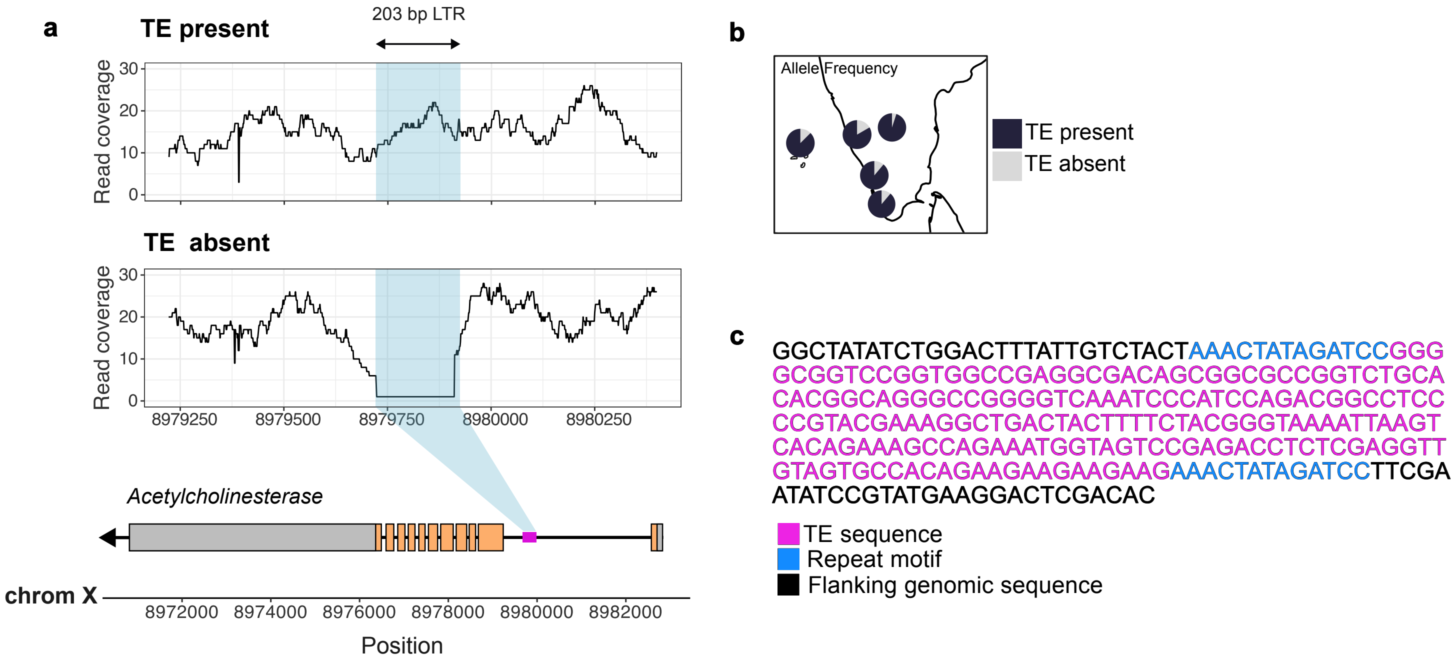

Supplementary Figure 8. **a.** Read coverage for a polymorphic TE insertion in the *Acetylcholinesterase-2* gene. The TE sequence is present in the UCI reference genome. Therefore, the absence of the TE insertion appears as a deletion that completely overlaps the TE fragment. This LTR retrotransposon fragment is inserted into the first intron of the gene. The length of the first intron suggests the presence of regulatory sites, which could be affected by the LTR fragment. **b.** The allele frequency of the TE fragment (1–deletion frequency) in the population samples. **c.** Nucleotide sequence from chrX:8,979,605–8,980,040 showing the LTR fragment flanked by a 13 bp repeat, which could result from a target site duplication when the TE inserted.

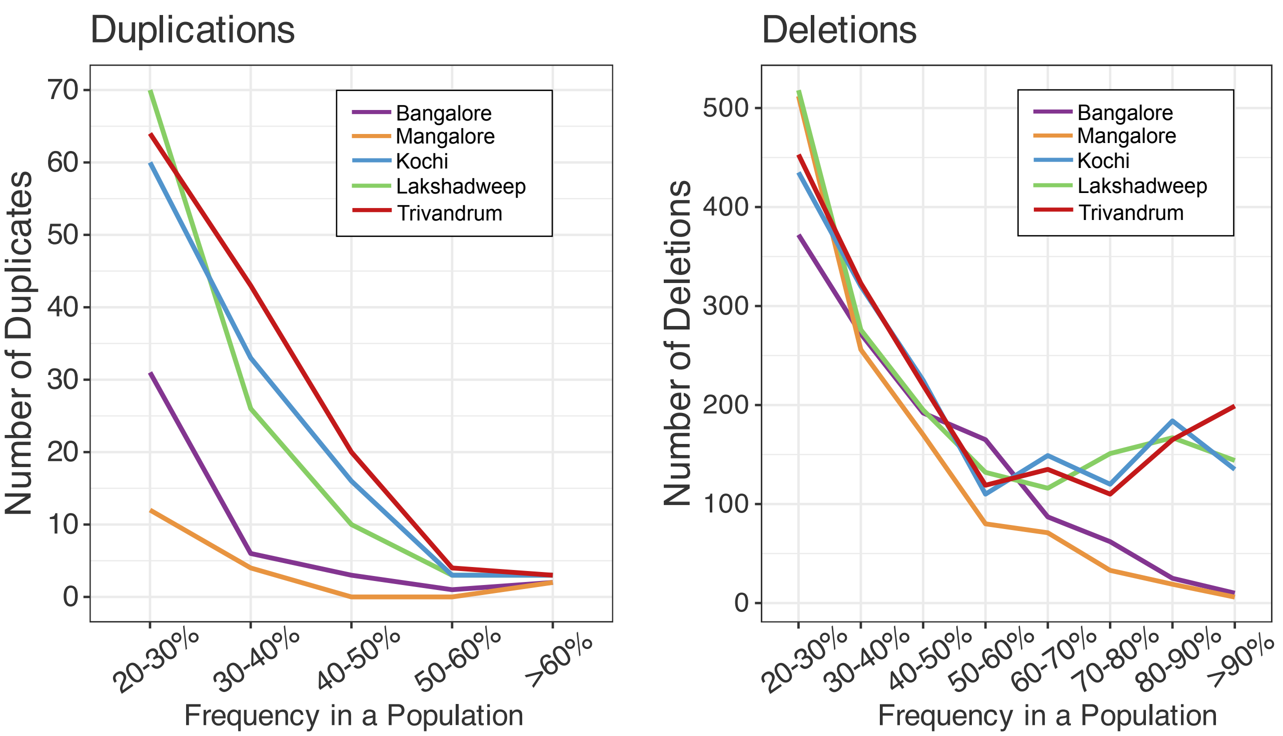

Supplementary Figure 9. Number of SVs segregating at frequencies greater than 20% in each population. The elevated number of deletions segregating at high frequencies in Kochi, Lakshadweep, and Trivandrum may include low-frequency insertions in the reference genome.

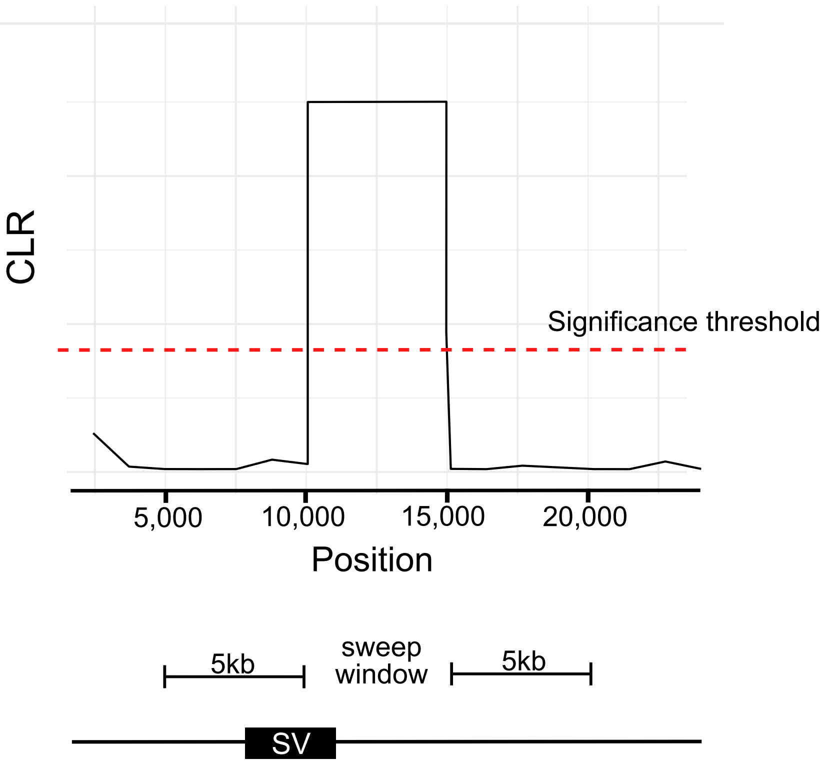

Supplementary Figure 10. Schematic demonstrating how it was determined whether an SV is associated with a CLR peak. The actual location of a sweep can be ~10 kbp from the CLR peak identified by SweepFinder2. Therefore, we included 5kbp on either side of the sweep window and overlapped it with our SV call set.

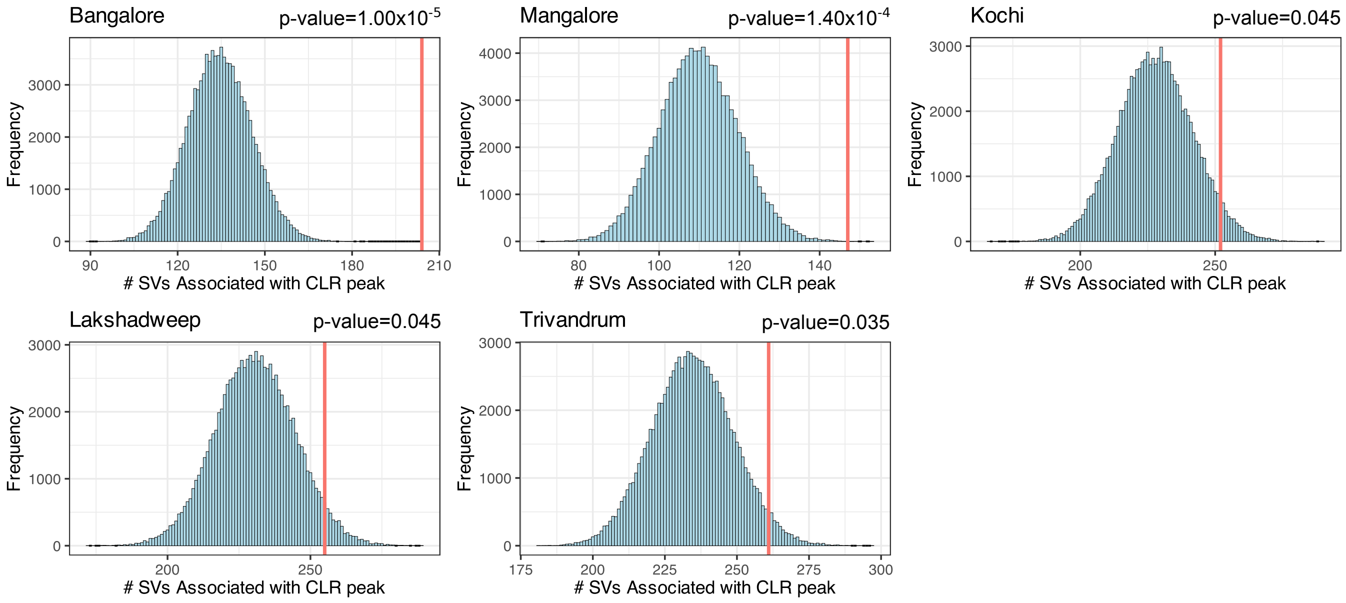

Supplementary Figure 11. Coordinates of SVs over 25% allele frequency in a population were shuffled 100,000 times. The number of SVs associated with CLR peaks was counted per run to generate null distributions for each population. Red lines represent the observed number of SVs over 25% allele frequency associated with CLR peaks.

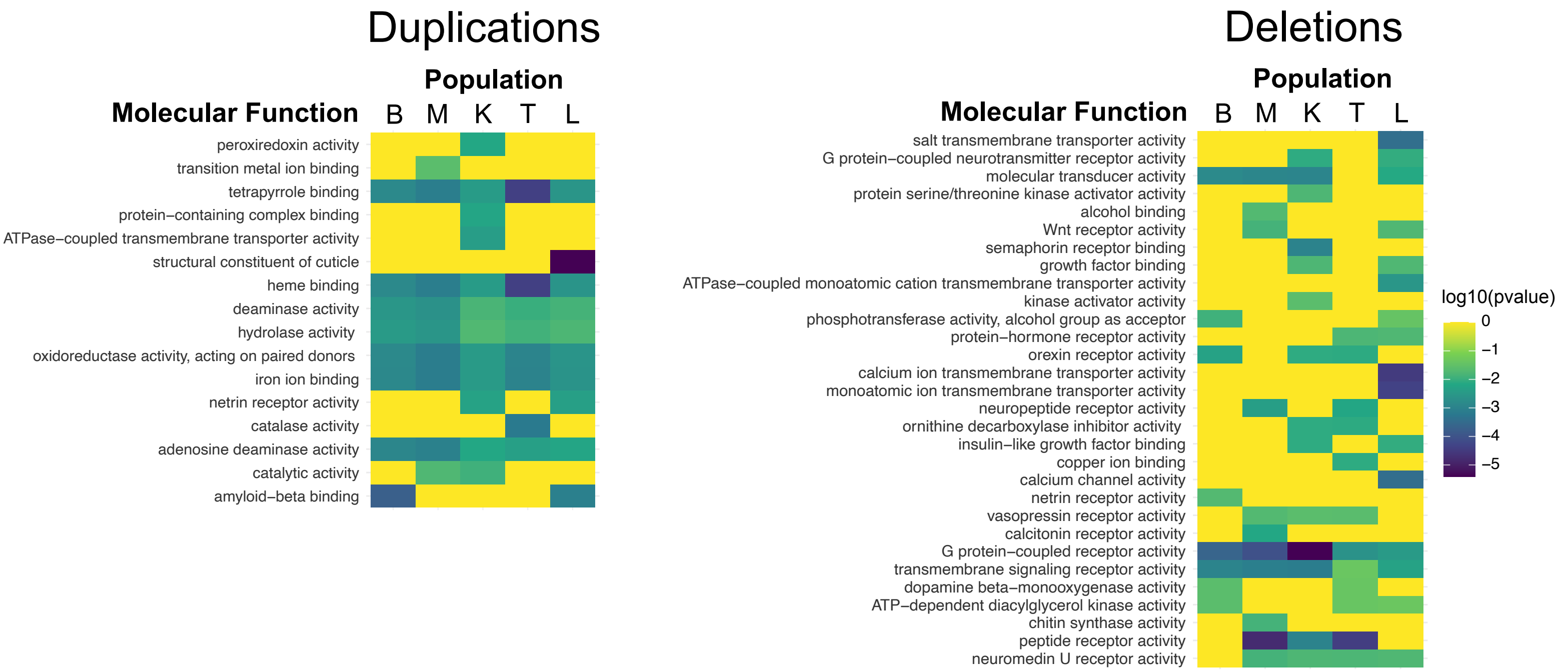

Supplementary Figure 12. GO term enrichment analysis for genes overlapped by duplications with allele frequency over 25% and associated with CLR peaks.

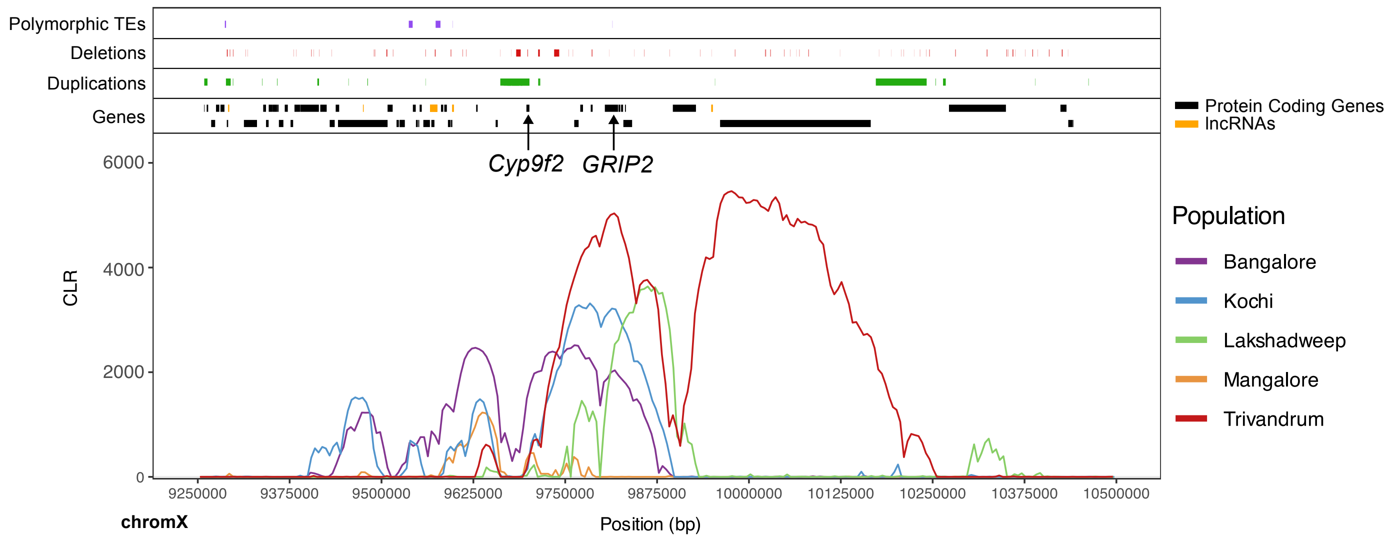

Supplementary Figure 13. SVs and annotated genes associated with prominent CLR peaks in all five populations examined. Most SVs in this region affect intergenic regions, thus, their functional significance is unclear. We highlight genes involved in insecticide resistance (*Cyp9f2*) and muscle guidance (*GRIP2*) which are overlapped by SVs.

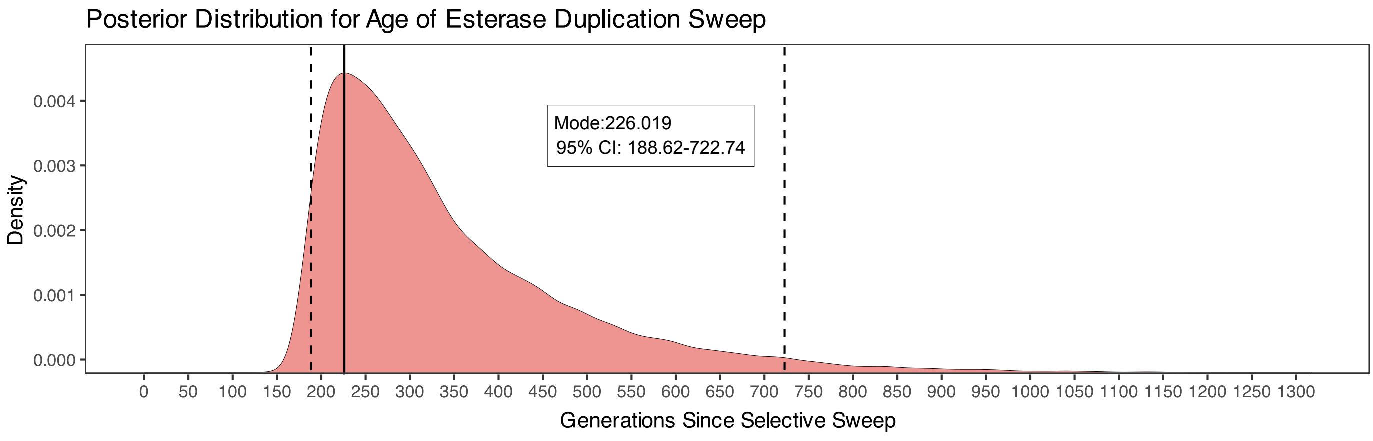

Supplementary Figure 14. The posterior probability distribution for the estimated age of a selective sweep in the Trivandrum population. This sweep is associated with a high-frequency duplication of carboxylesterases. The solid line marks the mode of the distribution, and the dotted lines mark 95% confidence intervals.

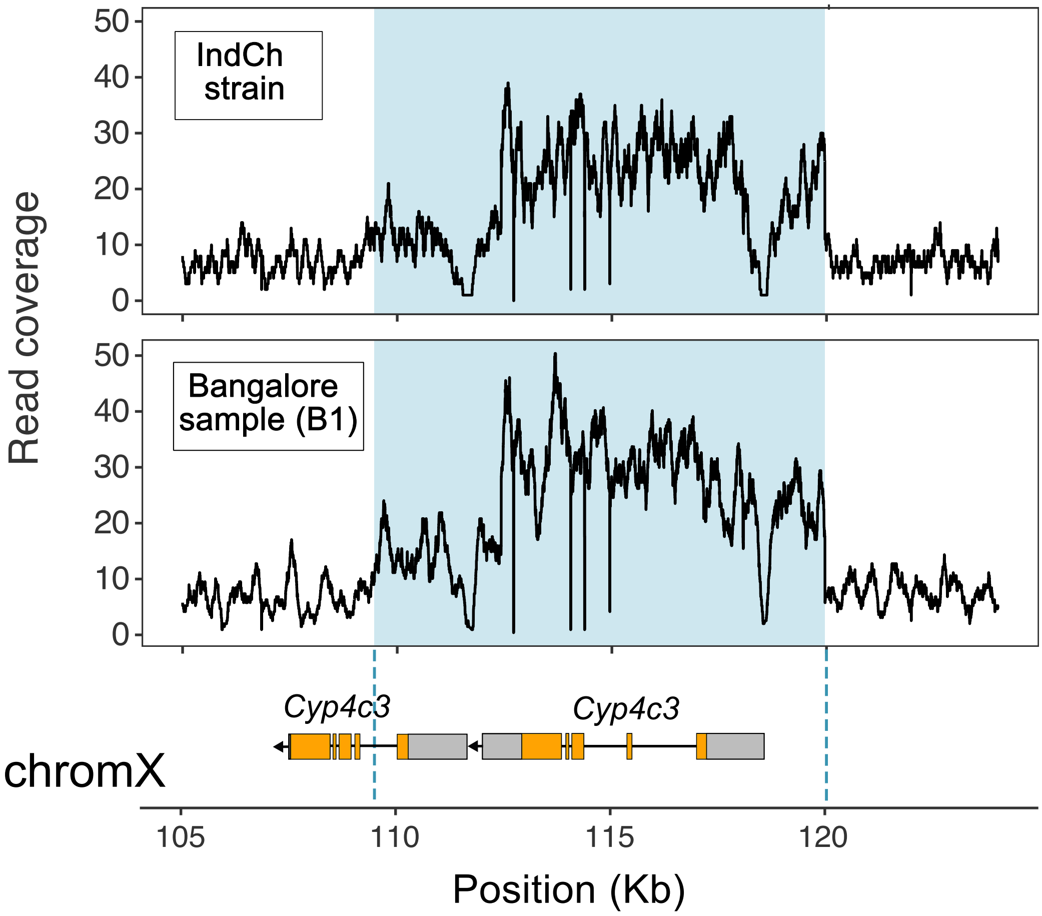

Supplementary Figure 15. Short-read coverage from *An. stephensi* IndCh strain (top) and a Bangalore wild sample (bottom) mapped to the UCI reference genome. Both samples show the same uneven coverage pattern at the *Cyp4C3* locus, suggesting that the fixed duplication described in this work may have the same complex duplication structure observed in the IndCh genome assembly.

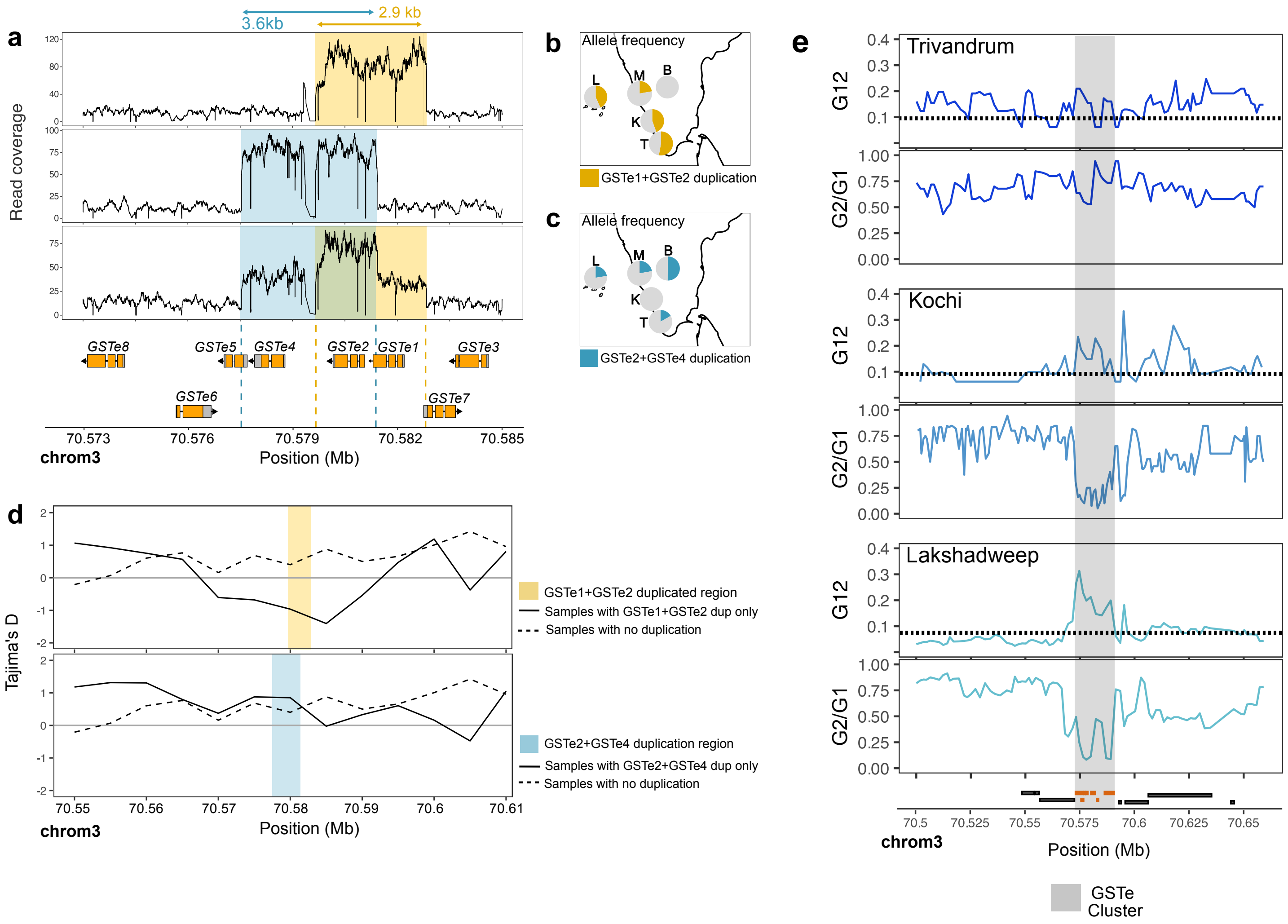

Supplementary Figure 16. a. Duplication CNVs in an array of epsilon Glutathione-S Transferase (GSTe) genes. One duplication (top) copies a sequence containing two full-length GSTes and partially overlaps another duplication allele (middle). 21 samples appear to have both alleles or a recombinant allele (bottom). **b.** Allele frequency of the duplication that copies *GSTe1* and *GSTe2*. **c.** Allele frequency of the duplication that copies *GSTe2, GSTe4.* **d.** Reduced Tajima's D flanking the *GSTe1+GSTe2* duplication indicates positive selection on the haplotype with only this duplication (top) but not in the haplotype with only the *GSTe2+GSTe+4* duplication. **e.** G12 and G2/G1 statistics in the Trivandrum, Kochi, and Lakshadweep population at the GSTe gene cluster (shaded region). Elevated G12 levels in this region, particularly in Kochi and Lakshadweep, suggest positive selection. A reduced G2/G1 ratio may indicate a hard sweep, whereas an elevated G2/G1 ratio aligns with a soft sweep, possibly driven by the presence of multiple duplication alleles.

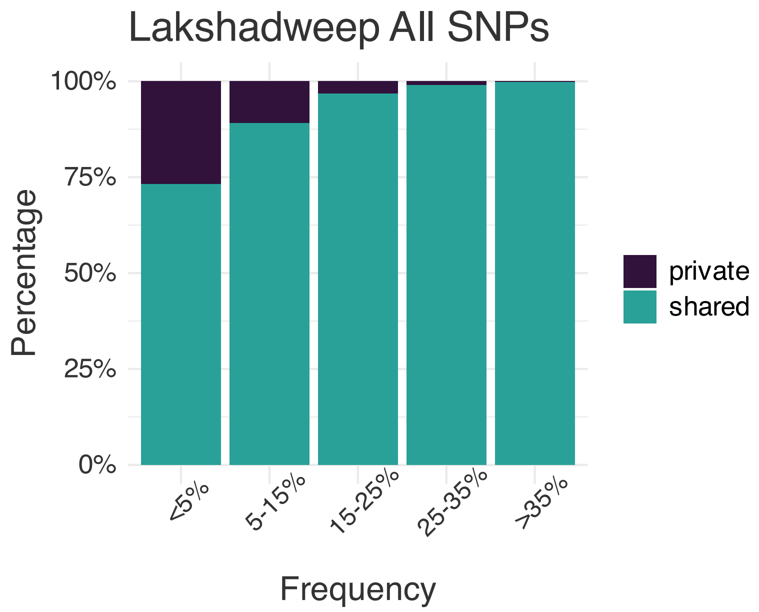

Supplementary Figure 17. The proportion of SNPs at different allele frequencies in the invasive island population that are private (found only on the island) or shared with at least one mainland population.

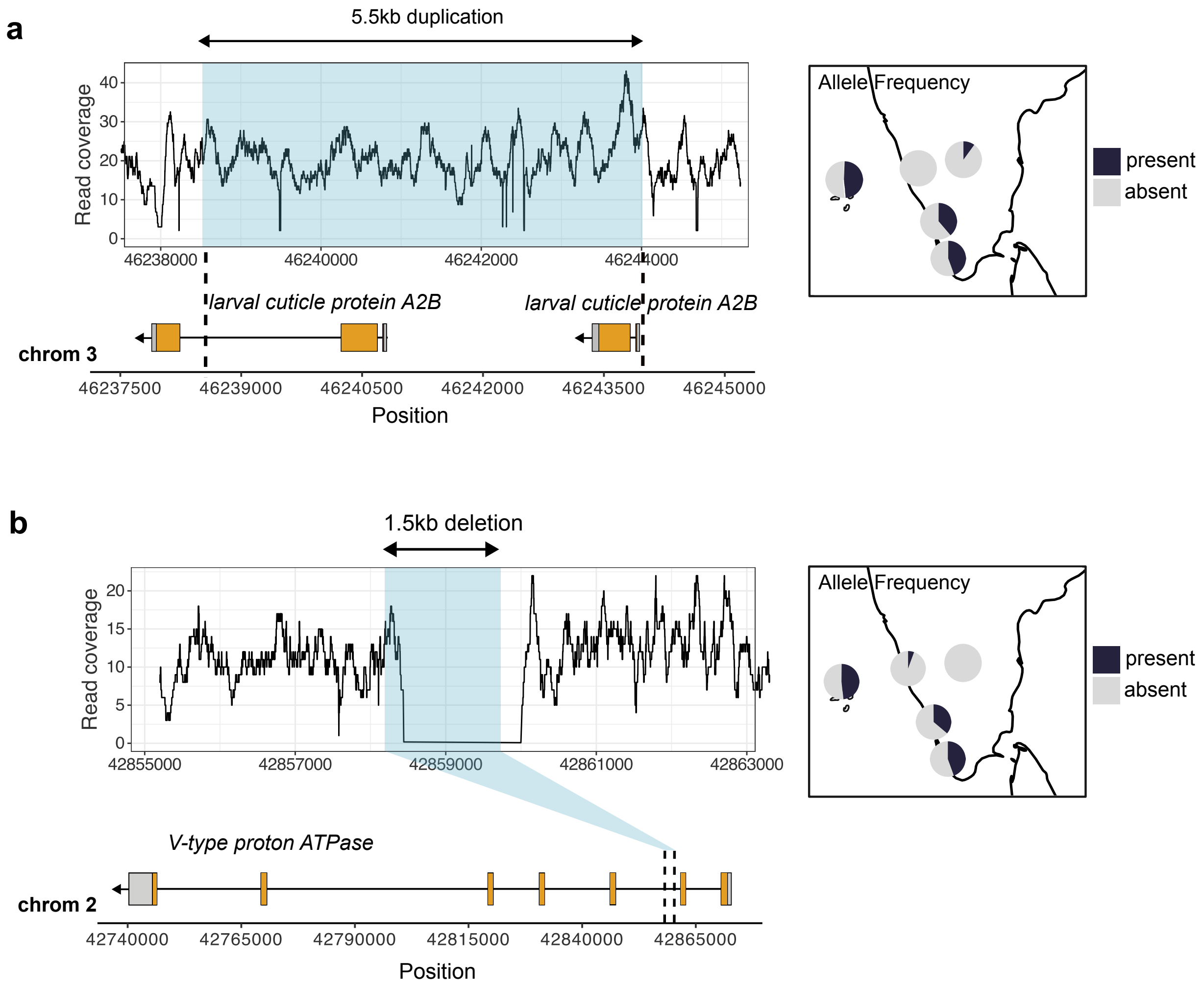

Supplementary Figure 18. Two candidate SVs found in coastal mainland and island populations. A duplication overlapping larval cuticle proteins (**a**) and an intronic deletion in an enzyme involved in osmoregulation (**b**) segregate at high frequencies in Kochi, Trivandrum, and Lakshadweep but are absent or low frequency in more inland populations.

Supplementary Tables

Supplementary Table 1. Sample IDs and coverage.

Supplementary Table 2. IndCh SVs validated using PacBio long read and 20x Illumina short read coverage.

Supplementary Table 3. SV genomic region enrichment/depletion analysis

	Region	Count	Fraction of SV Class	Mean Shuffled Fraction	p-value (Fisher's Exact Test)
**DUP**	intergenic	1171	0.392	0.355	0.003554
	whole gene	302	0.101	0.087	0.06913
	partial gene	584	0.195	0.255	3.45E-08
	intronic	820	0.274	0.264	0.3978
	exonic	111	0.037	0.039	0.8388
**DEL**	intergenic	8340	0.52	0.402	<2.20E-16
	whole gene	303	0.019	0.016	0.06082
	partial gene	646	0.04	0.015	<2.20E-16
	intronic	6179	0.385	0.355	1.18E-08
	exonic	570	0.036	0.092	<2.20E-16

Supplementary Table 4. SV genomic region distributions

	Region	Count	Fraction of all CNVs
**All DUPS and non-TE DELS**	intergenic	9511	0.5
	whole gene	605	0.032
	partial gene	1230	0.065
	intronic	6999	0.368
	exonic	681	0.036

Supplementary Table 5. Duplication CNV coordinates, allele frequency per population, associated CLR if above the 95 percentile, and gene overlap.

Supplementary Table 6. Deletion CNV coordinates, allele frequency per population, associated CLR if above the 95 percentile, and gene overlap.

Supplementary Table 7. Polymorphic reference TE coordinates, allele frequency per population (1 – deletion frequency), associated CLR if above the 95 percentile, and geneoverlap.

Supplementary Table 8. Missense SNPs associated with CLR above the 95 percentile in at least one population, allele frequency per population, and gene overlap.

Supplementary Table 9. Duplication and deletion CNV coordinates for variants segregating at allele frequency >0.25 and associated with a sweep window in Lakshadweep and their allele frequency in all five populations.
